# cAMP-mediated upregulation of HCN channels in VTA dopamine neurons promotes cocaine reinforcement

**DOI:** 10.1038/s41380-023-02290-x

**Published:** 2023-10-16

**Authors:** Lianwei Mu, Xiaojie Liu, Hao Yu, Casey R. Vickstrom, Vladislav Friedman, Thomas J. Kelly, Ying Hu, Wantang Su, Shuai Liu, John R. Mantsch, Qing-song Liu

**Affiliations:** 1 Department of Pharmacology and Toxicology, Medical College of Wisconsin, Milwaukee, WI 53226 USA; 2 Department of Exercise Physiology, Beijing Sport University, Beijing, 100084 China; 3 Present Address: Department of Neurology, Washington University School of Medicine, St. Louis, MO 63110 USA

**Keywords:** Neuroscience, Psychology

## Abstract

Chronic cocaine exposure induces enduring neuroadaptations that facilitate motivated drug taking. Hyperpolarization-activated cyclic nucleotide-gated (HCN) channels are known to modulate neuronal firing and pacemaker activity in ventral tegmental area (VTA) dopamine neurons. However, it remained unknown whether cocaine self-administration affects HCN channel function and whether HCN channel activity modulates motivated drug taking. We report that rat VTA dopamine neurons predominantly express *Hcn3-4* mRNA, while VTA GABA neurons express *Hcn1–4* mRNA. Both neuronal types display similar hyperpolarization-activated currents (I_h_), which are facilitated by acute increases in cAMP. Acute cocaine application decreases voltage-dependent activation of I_h_ in VTA dopamine neurons, but not in GABA neurons. Unexpectedly, chronic cocaine self-administration results in enhanced I_h_ selectively in VTA dopamine neurons. This differential modulation of I_h_ currents is likely mediated by a D_2_ autoreceptor-induced decrease in cAMP as D_2_ (*Drd2*) mRNA is predominantly expressed in dopamine neurons, whereas D_1_ (*Drd1*) mRNA is barely detectable in the VTA. Moreover, chronically decreased cAMP via Gi-DREADD stimulation leads to an increase in I_h_ in VTA dopamine neurons and enhanced binding of HCN3/HCN4 with tetratricopeptide repeat-containing Rab8b-interacting protein (TRIP8b), an auxiliary subunit that is known to facilitate HCN channel surface trafficking. Finally, we show that systemic injection and intra-VTA infusion of the HCN blocker ivabradine reduces cocaine self-administration under a progressive ratio schedule and produces a downward shift of the cocaine dose-response curve. Our results suggest that cocaine self-administration induces an upregulation of I_h_ in VTA dopamine neurons, while HCN inhibition reduces the motivation for cocaine intake.

## Introduction

Hyperpolarization-activated cyclic nucleotide-gated (HCN) channels are activated by membrane hyperpolarization and gated by cAMP [1, 2]. cAMP binding to a cyclic nucleotide binding domain (CNBD) of the HCN channel facilitates channel opening by shifting the threshold for activation to more depolarized membrane potentials and accelerating the kinetics of channel activation [3, 4]. cAMP-mediated modulation of HCN gating regulates many important processes; for example, β-adrenergic agonists increase heart rate by facilitating HCN activation in sinoatrial node cells [5, 6]; α_2A_-adrenergic tone in the prefrontal cortex strengthens working memory by decreasing cAMP gating of HCNs [7], and it reduces stress responses via the same mechanism in the bed nucleus of the stria terminalis [8]. cAMP modulation of HCN2 leads to neuronal hyperexcitability in nociceptors that transmit neuropathic pain [9]. These effects are mediated by acute neurotransmitter-mediated changes in cAMP and HCN gating. Much less is known about how chronic changes in cAMP affect HCN function. A recent study has shown that acute and chronic Gs-DREADD stimulation produced opposite modulation of HCN function in hippocampal CA1 pyramidal neurons via cAMP-dependent mechanisms [10]. Importantly, this mechanism may explain the delayed therapeutic effects of monoaminergic antidepressants, which are known to chronically elevate cAMP [10]. In addition, chronic increase in intracellular cAMP drives diabetes-associated pain by facilitating HCN2 activation and action potential (AP) firing in nociceptors [11].

Dopamine neurons in the ventral tegmental area (VTA) regulate reward, decision making, stress resilience and drug addiction [12, 13]. AP firing in VTA dopamine neurons is governed by voltage-dependent conductances including HCN [13–15]. Dopamine neurons express Gα_i/o_-coupled D_2_ dopamine autoreceptors that can be activated directly by dopamine or indirectly by cocaine [16–18]. Cocaine self-administration is expected to cause a chronic decrease in cAMP in dopamine neurons via D_2_ autoreceptor activation, however, it remains unknown whether cocaine self-administration affects HCN function and whether HCN activity modulates motivated cocaine taking. In the present study, we first conducted RNAscope to determine the expression of all four HCN isoforms (*Hcn1–4* mRNA) in VTA dopamine and GABA neurons. Next, we investigated whether bidirectional changes in cAMP alter I_h_ in identified VTA dopamine and GABA neurons. Thirdly, we examined whether cocaine self-administration alters I_h_ in both neuronal types and whether chronic decreases in cAMP mediates I_h_ modulation. Finally, we studied whether VTA-specific and systemic administration of ivabradine affects cocaine self-administration. Here, we demonstrate that cocaine self-administration led to an upregulation of I_h_ in VTA dopamine neurons via cAMP-dependent mechanisms, and both intra-VTA and systemic injections of ivabradine reduced the motivation to self-administer cocaine. Thus, HCN blockers may offer a potential therapeutic approach for the treatment of cocaine use disorder. This is particularly relevant considering that the most common cause for mortality from long-term cocaine use is dilated cardiomyopathy-associated heart failure [19], and that ivabradine is a HCN blocker that is FDA-approved for the management of chronic heart failure [20, 21].

## Materials and methods

### Animals

Long-Evans rats were purchased from Envigo (Indianapolis, IN). Heterozygous TH-Cre [22] and homozygous tdTomato reporter rats were obtained from the Rat Resource & Research Center (RRRC, Columbia, MO) and were crossed to produce TH-tdTomato reporter rats. Roughly equal numbers of male and female rats (7–9-week-old at the beginning of experiments) were used. All protocols were approved by the Medical College of Wisconsin’s Institutional Animal Care and Use Committee.

### RNAscope in situ hybridization

Following transcardial perfusion with 4% paraformaldehyde, rat brains were extracted and rapidly frozen on dry ice. Coronal midbrain sections (15 μm) were cut on a cryostat (Leica CM1860, Nussloch, Germany). Fluorescent probes targeting *Rattus norvegicus* mRNA (Advanced Cell Diagnostics Inc; Hayward, CA) were incubated with the sections per the manufacturer’s directions and imaged using a Leica TCS SP8 confocal microscope. RNAscope was performed as described [23, 24]. The relative expression levels of mRNA that encode *Slc6a3*, *Gad1*, *Hcn1*, *Hcn2*, *Hcn3*, *Hcn4*, *Drd1* and *Drd2* in the VTA were quantified and compared using Imaris (Bitplane, Zürich, Switzerland).

### Slice preparation and electrophysiology

Rats were anesthetized with isoflurane and transcardially perfused with NMDG-based solution [25, 26]. Brains were removed, trimmed, and embedded in low-gelling-point agarose. Horizontal VTA slices (200 μm) were cut using a vibrating slicer (Leica VT1200s) in NMDG-based solution [25, 26]. Na^+^ was gradually reintroduced [25, 26] and slices were allowed to rest in ACSF for at least an additional 30 min. Whole-cell recordings and protocols to measure I_h_ currents were conducted as described [27, 28].

### Stereotaxic surgeries, AAV injections, and jugular catheterization

Prior to surgeries, rats were anesthetized with ketamine (90 mg/kg, i.p.) and xylazine (10 mg/kg, i.p.). For jugular catheterization, a round-tip polyurethane catheter (C30PU-RJV1611, Instech Laboratories, Inc, Plymouth Meeting, PA) was inserted into the right jugular vein. The catheter was connected to a vascular access button (22-gauge; VABR1B/22, Instech) and implanted subcutaneously on the back of the rats. Intravenous catheters were flushed with 0.2 ml of heparinized saline (30 units/ml) and cefazolin (100 mg/ml), and patency was tested by i.v. infusion of 0.05 ml xylazine (20 mg/ml) weekly or when a compromised catheter was suspected [29]. For stereotaxic surgery, rats were placed into Neurostar stereotaxic devices (Neurostar, Tübingen, Germany). For intra-VTA microinjections, guide cannulae (26 gauge; P1 Technologies, Roanoke, VA) were implanted 2.8 mm above the VTA at stereotaxic coordinates: AP, −5.3 mm; ML ± 2.4 mm; DV, −7.8 mm; 10° angle [30, 31]. For DREADD experiments, a glass capillary Nanoinjector (Neurostar) and a Nanoject III Programmable Nano-liter Injector (Drummond Scientific Company, Broomall, PA) were used to inject AAV8-hSyn-DIO-hM4D(Gi)-mCherry or AAV8-hSyn-DIO-mCherry (250 nl each; Addgene, Watertown, MA) into the VTA of TH-Cre rats at the same coordinates. Rats received an analgesic injection (buprenorphine-SR, 1 mg/kg, s.c.) immediately following surgery. Rats were allowed for recovery of 1–2 weeks prior to the start of self-administration experiments.

### Cocaine self-administration and yoked administration

Cocaine self-administration was conducted similarly to our previously published studies [25, 32]. Rats with no prior lever press training were placed into operant conditioning chambers (Med Associates Inc., Fairfax, VT) and allowed to self-administer cocaine for 10 days in 3 h sessions. Active responses resulted in a cocaine infusion and illumination of a cue light above the active lever for 5 s, followed by a 10 s timeout during which active responses were recorded but did not result in further infusions. Responding was maintained at fixed ratio 1 (FR1, 1 mg/kg/infusion) on days 1–5 and fixed ratio 2 (FR2, 0.5 mg/kg/infusion) on days 6–10. Rats that did not acquire stable self-administration after 10 days were excluded. Timepoints of cocaine infusions from rats that successfully acquired stable self-administration were used to yoke the delivery of i.v. saline, cocaine, or deschloroclozapine (DCZ) in additional cohorts of rats. One day following self-administration or yoked administration, slices were prepared for patch-clamp electrophysiology, as described above.

Additional cohorts of rats were used for testing the effects of intra-VTA infusion or systemic injection of ivabradine on FR2, FR2 multiple-dose and progressive ratio (PR) schedules. For intra-VTA infusion of ivabradine, the day following completion of self-administration training, vehicle (0.5 μl/side) or ivabradine (25 or 50 ng/0.5 µl/side) were bilaterally microinjected into the VTA via injector cannulae (33-gauge; P1 Technologies, Roanoke, VA). Cocaine self-administration was tested 10 min later. For i.p. ivabradine, rats first received i.v. injection of elacridar (5 mg/kg) followed by i.p. injection of ivabradine (3 or 10 mg/kg) or vehicle (0 mg/kg). Cocaine self-administration was tested 10 min later.

### Co-Immunoprecipitation (Co-IP) reactions

Rats were deeply anesthetized with isoflurane, decapitated, and the coronal midbrain was cut using a rat brain matrix. Bilateral VTA was punched out and flash frozen. Co-IP reactions were performed with a commercial kit (26149, ThermoFisher, Rockford, IL) using anti-HCN3 (APC-057) or anti-HCN4 antibodies (APC-052, both from Alomone labs, Jerusalem, Israel).

### Chemical reagents

ZD7288, (RS)-(±)-sulpiride, rolipram, forskolin and ivabradine hydrochloride were purchased from Tocris Bioscience (Ellisville, MO). DCZ dihydrochloride (water soluble) was purchased from Hello Bio Inc. (Princeton, NJ). Elacridar hydrochloride was purchased from Medchemexpress LLC (Monmouth Junction, NJ). Cocaine HCl was provided by the NIDA Drug Supply Program. All other common chemicals were obtained from Sigma-Aldrich (St. Louis, MO).

### Statistics

Data are presented as the mean ± SEM. Animal numbers and sample sizes are calculated based on statistical power analysis (α = 5%, Power = 0.8). For behavioral tests, prior to statistical testing equal variance was determined with Levene’s test, normality was assessed with the Shapiro-Wilk test using OriginPro. Data sets were compared with either Student’s *t*-test, Paired *t*-test, one-way ANOVA followed by Tukey’s *post hoc* analysis, two-way repeated measures (RM) ANOVA, Kruskal-Wallis one-way ANOVA on ranks followed by Dunn’s *post hoc* analysis for pair-wise comparisons, or the Kolmogorov-Smirnov test (K-S test). *Post hoc* analyses were performed only when ANOVA yielded a significant main effect or a significant interaction between the two factors. Results were significant at *p* < 0.05.

## Results

### Differential expression of *Hcn1–4* mRNA in VTA dopamine and GABA neurons

**Fig. 1:**
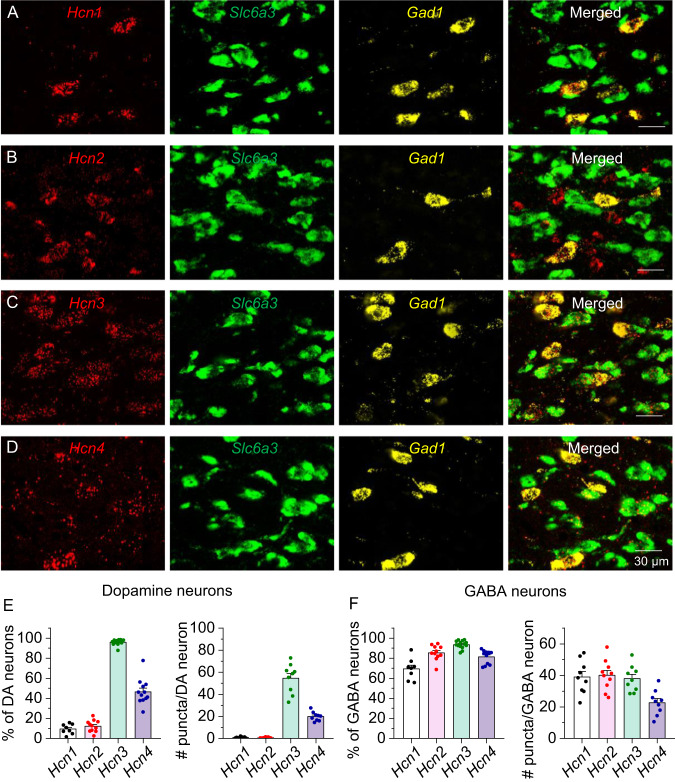
Expression of *Hcn1–4* mRNA in rat VTA dopamine and GABA neurons. Representative 63x images of VTA sections labeled with RNAscope in situ hybridization for *Hcn1–4* (red), *Gad1* (GABA neuron, yellow) and *Slc6a3* (dopamine neuron, green) mRNA. **A**, **B** *Hcn1* and *Hcn2* mRNA were highly expressed in *Gad1* neurons, but barely detectable in *Slc6a3* neurons. *Hcn2* mRNA was also expressed on a cell type that remains to be detected. **C**, **D** *Hcn3* and *Hcn4* mRNA were expressed in both *Gad1* neurons and *Slc6a3* neurons. **E** Left, percentage of VTA dopamine neurons co-expressing *Hcn1–4* (*Hcn1*: 208 of 2245 neurons; *Hcn2*: 286 of 2350 neurons; *Hcn3*: 1591 of 1661 neurons; *Hcn4*: 1109 of 2392 neurons). Right, the number of *Hcn1–4* mRNA puncta expressed in VTA dopamine neurons. **F** Left, percentage of VTA GABA neurons co-expressing *Hcn1–4* (*Hcn1*: 428 of 619 neurons; *Hcn2*: 628 of 736 neurons; *Hcn3*: 509 of 545 neurons; *Hcn4*: 514 of 634 neurons. Right, the number of *Hcn1–4* mRNA puncta expressed in VTA GABA neurons. (*n* = 8 to 14 imaged sections from 4 rats).

HCN channels are assembled predominantly as heterotetramers from four subunits encoded by *Hcn1–4* [1, 33, 34]. Brain-wide in situ hybridization has shown that *Hcn1–4* mRNA is expressed in the VTA in adult rat brain [35]. Immunohistochemistry has shown that HCN2 is expressed in the rat VTA [36], and HCN4 is expressed in VTA dopamine and GABA neurons [37]. However, previous studies have not directly compared the expression of HCN channel isoforms in specific VTA cell types systematically. We conducted RNAscope in situ hybridization to assess the cell-type distribution of all four *Hcn* isoforms in the VTA. *Hcn1* and *Hcn2* mRNA were expressed in 69.3 ± 3.7% and 85.3 ± 2.2% GABA neurons, respectively, as shown by colocalization with glutamic acid decarboxylase 1 (*Gad1*) mRNA, but these were rarely expressed in dopamine neurons (*Hcn1*, 9.3 ± 1.7%; *Hcn2*,12.2 ± 1.8%) as defined by colocalization with solute carrier family 6 member 3 (*Slc6a3*, dopamine transporter, DAT) mRNA (Fig. 1A, B, E, F, Figs. S1, S2). *Hcn3* mRNA was expressed in 93.4 ± 1.1% GABA and 95.8 ± 0.7% dopamine neurons, *Hcn4* mRNA was expressed in 81.2 ± 1.9% GABA and 46.4 ± 3.7% dopamine neurons (Fig. 1C–F, Figs. S3, S4). Thus, VTA dopamine neurons express mainly *Hcn3-4* mRNA, while VTA GABA neurons express all four *Hcn* isoforms. Detailed statistics for this and subsequent results are described in figure legends.

### I_h_ currents in VTA dopamine and GABA neurons are sensitive to cAMP

**Fig. 2:**
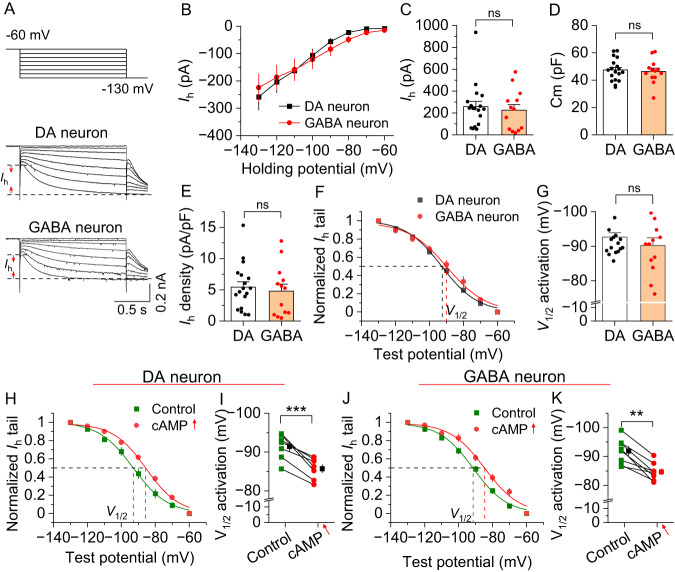
I_h_ currents were not significantly different between VTA dopamine and GABA neurons and were sensitive to cAMP stimulation. **A** Left: Voltage protocol for recording I_h_ current. Right: Representative I_h_ traces recorded from dopamine (DA) neurons and GABA neurons in the VTA. **B** There was no significant difference in I_h_ between dopamine and GABA neurons at all corresponding hyperpolarization potentials (two-way RM ANOVA, cell-type, *F*_1,29_ = 0.02, *p* = 0.894; holding potential, *F*_7,203_ = 37.7, *p* < 0.001; cell-type ⨯ holding potential interaction, *F*_7,203_ = 0.6, *p* = 0.770; DA neuron, *n* = 18 cells; GABA neuron, 13 cells; *n* = 4 rats). **C** I_h_ amplitude was calculated by subtracting the instantaneous current from the steady-state current achieved during the voltage step at −130 mV, and no significant difference was detected (*t*-test, *t*_29_ = 0.5, *p* = 0.640, *n* = 13–18 cells). **D** There was no significant difference in membrane capacitance (C_m_) between dopamine and GABA neurons (*t*-test, *t*_29_ = 0.4, *p* = 0.728, *n* = 13–18). **E** I_h_ current density (=I_h_ amplitude at −130 mV/C_m_) was not significantly different between dopamine neurons and GABA neurons (*t*-test, *t*_29_ = 0.5, *p* = 0.655, *n* = 13–18). **F** I_h_ activation curves in dopamine neurons and GABA neurons were generated by the tail current protocol. Tail current amplitudes were fitted with a Boltzmann function. **G** There was no significant difference in the midpoint activation voltage (*V*_1/2_) between dopamine and GABA neurons (*t*-test, *t*_29_ = 1.0, *p* = 0.322). **H**, **I** Increasing cAMP (cAMP ↑ ) via bath perfusion of forskolin (20 µM) and rolipram (1 µM) led to a significant rightward shift in the I_h_ activation curve of and a significant depolarizing shift in the *V*_1/2_ of VTA dopamine neurons (paired *t*-test *t*_6_ = 6.4, *p* < 0.001, *n* = 7 from 3 rats). **J**, **K** Increasing cAMP led to a significant depolarizing shift in the I_h_ activation curve and a depolarizing shift in the *V*_1/2_ of VTA GABA neurons (paired *t*-test, *t*_7_ = 3.9, *p* = 0.006, *n* = 8 from 3 rats). ns, not significant, *p* > 0.05, **p < 0.01, ***p < 0.001.

Among HCN1–4, HCN2 and HCN4 are most sensitive to cAMP, while HCN1 and HCN3 show less cAMP sensitivity [3, 38, 39]. We recorded HCN currents (I_h_) in identified dopamine and GABA neurons in the VTA. We labeled midbrain dopamine neurons with fluorescent reporter tdTomato by breeding tyrosine hydroxylase (TH)-Cre rats [22] with tdTomato reporter rats. Consistent with the previous study [22], all tdTomato^+^ neurons colocalized with TH, but only 64.7 ± 1.8% TH^+^ neurons expressed tdTomato (Fig. S5). Thus, TH-tdTomato labeling of dopamine neurons is specific but incomplete. As tdTomato-negative neurons consisted of both GABA and dopamine neurons, we identified these neurons by collecting cytoplasmic mRNA for single-cell RT-PCR upon the completion of whole-cell recordings (Fig. S6). I_h_ currents were recorded as described [27]. Consistent with previous studies [37, 40], there were no significant differences of the I_h_ amplitude, membrane capacitance (C_m_), and I_h_ current density and midpoint activation voltage (*V*_1/2_) between VTA dopamine and GABA neurons (Fig. 2A–G). I_h_ currents in both dopamine neurons and GABA neurons were abolished by HCN blockers ZD7288 (20 μM) and ivabradine (50 μM) (data not shown). Bath application of the adenylyl cyclase activator forskolin (20 μM) and the phosphodiesterase-4 (PDE4) inhibitor rolipram (1 μM), which increased intracellular cAMP in the VTA [30, 41], induced similar depolarizing shifts of *V*_1/2_ of I_h_ in VTA dopamine (Fig. 2H, I) and GABA neurons (Fig. 2J, K). These results are consistent with the expression of cAMP-sensitive HCN isoforms in both VTA dopamine (*Hcn4*) and GABA neurons (*Hcn2/Hcn4*) as revealed by RNAscope.

### Acute cocaine application modulated I_h_ in VTA dopamine neurons via D_2_ autoreceptors

**Fig. 3:**
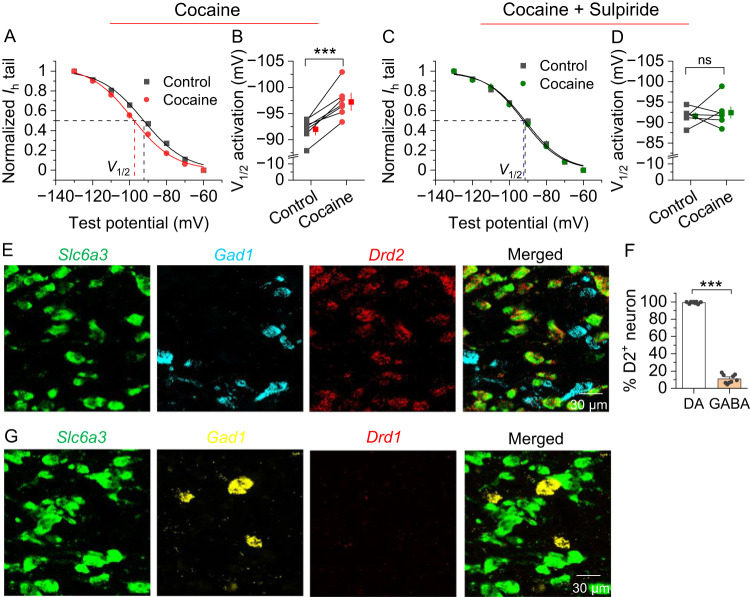
Bath application of cocaine induced a hyperpolarizing shift of the *V*_1/2_ of I_h_ currents in VTA dopamine neurons through activation of the D_2_-autoreceptor. **A**, **B** Bath perfusion of cocaine (10 μM) induced a significant leftward shift in the I_h_ activation curve and a hyperpolarizing shift of the *V*_1/2_ of VTA dopamine neurons (paired *t*-test, *t*_6_ = 6.3, *p* < 0.001, *n* = 7 from 3 rats). **C**, **D** The shifts of cocaine on the I_h_ activation curve and the *V*_1/2_ were blocked by D_2_ receptor antagonist sulpiride (1 μM) (paired *t*-test, *t*_5_ = 0.5, *p* = 0.641, *n* = 6 from 3 rats). **E**, **F** *Drd2* mRNA was abundantly expressed and completely colocalized with *Slc6a3* mRNA but was expressed only in ~10% of GABA neurons (ns, not significant, *p* > 0.05, ***p < 0.001; *n* = 2 rats). **G** *Drd1* mRNA was barely detected in both dopamine and GABA neurons in the VTA (*n* = 2 rats).

We next determined whether decreasing cAMP via Gα_i/o_-coupled dopamine D_2_ receptors alters I_h_ currents in VTA dopamine neurons and GABA neurons. D_2_ receptors are coupled to G protein-coupled inwardly rectifying potassium channels (GIRK) in midbrain dopamine neurons [42]. In the presence of the GIRK channel blocker BaCl_2_ (300 μM), bath application of cocaine (10 μM) produced a hyperpolarizing shift of *V*_1/2_ in VTA dopamine neurons (Fig. 3A, B), which was blocked by the D_2_ receptor antagonist sulpiride (1 μM) (Fig. 3C, D). Cocaine did not significantly alter I_h_ in VTA GABA neurons (Fig. S7). RNAscope showed that dopamine receptor D_2_ (*Drd2*) mRNA was expressed in virtually all dopamine neurons but only in 10.7 ± 1.7% GABA neurons (Fig. 3E, F, Fig. S8), which may explain why cocaine produced differential modulation of I_h_ in dopamine and GABA neurons. In contrast, D_1_ (*Drd1*) mRNA was barely detectable in the VTA (Fig. 3G, Fig. S9) but *Drd1* mRNA clusters were detected in regions neighboring to the VTA (Fig. S9); In addition, *Drd1* and *Drd2* mRNA were abundantly expressed in the striatum with minimal overlap (3.5 ± 0.7%) (Fig. S10).

### Chronic intravenous cocaine administration altered I_h_ currents and temporal summation of EPSPs in VTA dopamine neurons

**Fig. 4:**
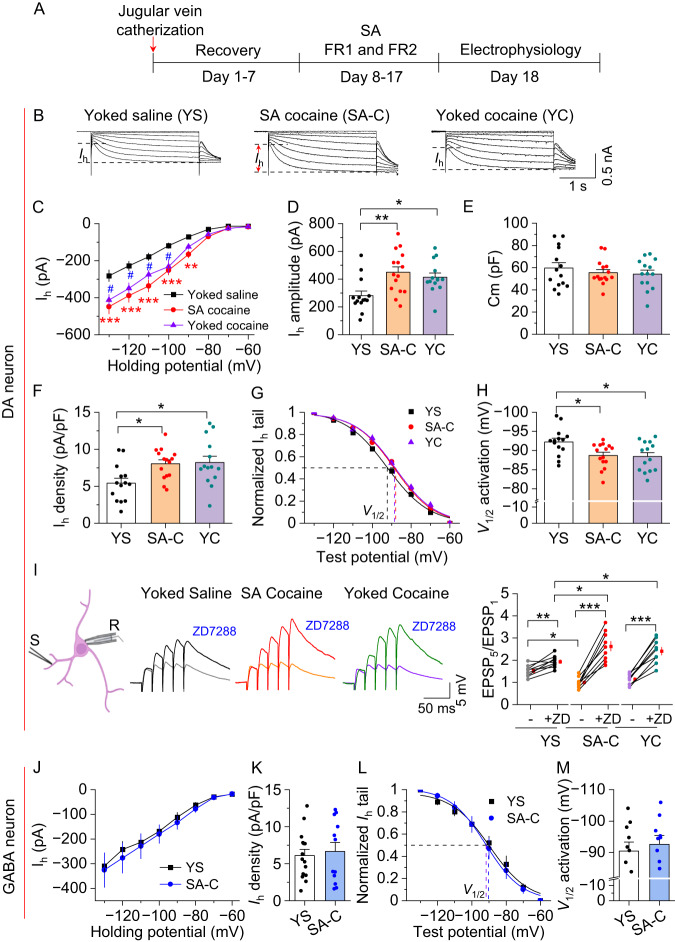
Cocaine self-administration led to upregulation of I_h_ in VTA dopamine neurons. **A** Timeline of jugular vein catheterization, cocaine self-administration and electrophysiology. **B** Representative I_h_ currents recorded from VTA dopamine neurons after 10 days of yoked saline (YS), cocaine self-administration (SA) and yoked cocaine infusions (YC). **C** Compared with yoked saline infusions, cocaine self-administration and yoked cocaine infusions led to significant increases in I_h_ currents at corresponding hyperpolarization potentials (two-way RM ANOVA, Cocaine, *F*_2,40_ = 8.6, *p* < 0.001; holding potential, *F*_7,280_ = 248.9, *p* < 0.001; Cocaine ⨯ holding potential interaction, *F*_14,280_ = 5.5, *p* < 0.001; *n* = 14–15 from 5 rats; yoked saline vs cocaine SA, blue * and ^#^, yoked saline vs yoked cocaine, red * and ^#^; *p < 0.01, ^#^*p* < 0.001). **D**–**F** Cocaine self-administration and yoked cocaine infusions led to significant increases in I_h_ amplitude (**D**; one-way ANOVA, *F*_2,42_ = 6.5, *p* = 0.004, *n* = 14-15) and I_h_ density (**F**; one-way ANOVA, *F*_2,42_ = 5.1, *p* = 0.010, *n* = 14–15 cells from 5 rats), but did not significant change the membrane capacitance (C_m_) of VTA dopamine neurons (**E**; one-way ANOVA, *F*_2,42_ = 0.5, *p* = 0.587, *n* = 14–15 cells). **G**, **H** Cocaine self-administration and yoked cocaine infusions led to depolarizing shifts in the I_h_ activation curve the *V*_1/2_ of VTA dopamine neurons (one-way ANOVA, *F*_2,42_ = 4.9, *p* = 0.012, *n* = 14–15 cells). **I** Left: A schematic diagram shows the location of the stimulating electrode (S) and recording electrode (R). Representative EPSPs (50 Hz ⨯ 5) showing temporal summation before and after application of ZD7288. Right, Comparison of changes in temporal summation of EPSPs (EPSP5/EPSP1 ratio) in cocaine self-administration, yoked cocaine and yoked saline groups in the absence and presence of ZD7288 (two-way RM ANOVA, Cocaine, *F*_2,24_ = 0.2, *p* = 0.856; ZD7288, *F*_1,24_ = 172.0, *p* < 0.001; Cocaine ⨯ ZD7288 interaction, *F*_1,24_ = 18.1, *p* < 0.001; *n* = 9 cells from 4 rats). **J**–**M** Cocaine self-administration did not alter the I_h_ density (**J**, **K**; *t*-test, *t*_25_ = 0.4, *p* = 0.716) and *V*_1/2_ (**L**, **M**; *t*-test, *t*_18_ = 0.5, *p* = 0.597) of GABA neurons in the VTA. For (**D**–**I**), *p < 0.05, **p < 0.01, ***p < 0.001.

We next investigated whether cocaine self-administration altered I_h_ currents in VTA dopamine and GABA neurons, which enabled us to study how chronic changes in cAMP alter HCN function. Rats were trained to self-administer cocaine for 10 days, and separate groups of rats received saline or cocaine infusions yoked to the infusion schedule of previously self-administering rats. To determine whether cocaine self-administration decreases cAMP levels in the VTA, we used ELISA to measure cAMP levels from VTA tissue punches immediately after the last saline or cocaine administration. We found that cAMP levels in the VTA were decreased in rats that received yoked cocaine administration or cocaine self-administration compared to those in saline-administered rats (Fig. S11). Next, TH-tdTomato rats were trained for cocaine self-administration, yoked cocaine or saline administration for 10 days. Midbrain slices were prepared 1 day after the last cocaine or saline infusion (Fig. 4A). Compared with those of the yoked saline group, cocaine self-administration and cocaine yoked infusions led to significant increases in the amplitude and current density of I_h_ in VTA dopamine neurons (Fig. 4B–D, F). The changes in I_h_ in dopamine neurons cannot be attributed to a decrease in cell size, as membrane capacitance (C_m_, Fig. 4E) and surface area of dopamine neurons (as determined by TH immunohistochemistry, Fig. S12) were not significantly different between the cocaine group and the yoked saline group. Cocaine self-administration and yoked cocaine infusions also produced a depolarizing shift of *V*_1/2_ in VTA dopamine neurons (Fig. 4G, H). In contrast, cocaine self-administration did not change I_h_ amplitude, I_h_ density and *V*_1/2_ in VTA GABA neurons compared with the yoked saline group (Fig. 4J–M).

HCN activation at dendrites limits temporal summation of excitatory postsynaptic potentials (EPSPs) [10, 43, 44]. We next determined whether the cocaine-induced increase in I_h_ currents were associated with changes in temporal summation of EPSPs. EPSPs were evoked by a train of electrical stimulation (50 Hz ⨯ 5) of excitatory synaptic inputs in the presence of a cocktail of GABA_A_, GABA_B_, D_2_ and NMDA receptor antagonists. It is worth noting that VTA dopamine neurons typically exhibit tonic and bursting activity, with the latter characterized by phasic firing at a frequency range of 15–30 Hz [45]. This phasic firing can reach up to 60 Hz in stressed animals [46]. However, the choice of the 50 Hz stimulation frequency was not intended to mimic the in vivo firing patterns; rather, this high frequency stimulation has been commonly utilized in the assessment of the temporal summation of EPSPs, which indirectly reflects the strength of dendritic I_h_ [10, 43, 44]. Consistent with increased I_h_, we found that VTA dopamine neurons in the cocaine self-administration and yoked cocaine groups exhibited significantly less temporal summation of evoked EPSPs compared with the yoked saline control, and ZD7288 induced a greater increase in temporal summation of EPSPs in the cocaine-exposed groups than that in the yoked saline group (Fig. 4I).

HCN channels are important in governing pacemaker activity in midbrain dopamine neurons [13–15]. We examined whether cocaine self-administration was associated with changes in spontaneous AP firing in VTA dopamine neurons and whether ZD7288 affected AP firing. We found that cocaine self-administration led to a significant increase in spontaneous AP firing in VTA dopamine neurons compared to yoked saline administration, and ZD7288 produced a greater suppression of spontaneous AP firing in cocaine group than that in saline group (Fig. S13). Thus, cocaine self-administration is accompanied by an increase in the activity of VTA dopamine neurons, ZD7288 reduces AP firing and abrogates the difference in AP firing between cocaine and saline groups.

### Chronically decreased cAMP induced an up-regulation of I_h_ in VTA dopamine neurons

**Fig. 5:**
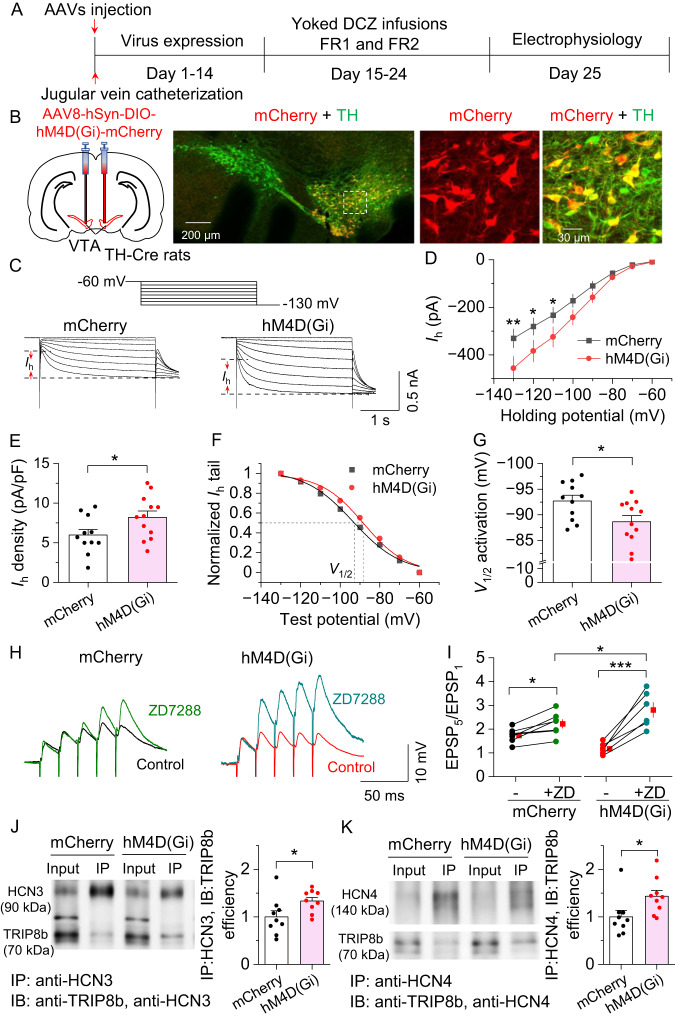
Chronically decreasing cAMP with hM4D(Gi) induced an up-regulation of I_h_ in VTA dopamine neurons. **A** Timeline of viral injection, yoked DCZ infusions and electrophysiology. **B** AAV8-hSyn-DIO-hM4D(Gi)-mCherry [hM4D(Gi)] or AAV8-hSyn-DIO-mCherry (mCherry) was bilaterally microinjected into the VTA of TH-Cre rats. Immunohistochemistry showed that hM4D(Gi) was expressed in the majority of TH^+^ dopamine neurons (green) but was not expressed in TH^-^ neurons in the VTA of TH-Cre rats (*n* = 5 rats). **C** Representative I_h_ currents recorded from rats that received yoked DCZ infusions expressing mCherry or hM4D(Gi) in VTA dopamine neurons. **D** I_h_ amplitude was significantly increased in rats with expressing hM4D(Gi) compared to mCherry at the corresponding hyperpolarization potentials (two-way RM ANOVA, hM4D(Gi), *F*_1,21_ = 2.7, *p* = 0.117; holding potential, *F*_7,147_ = 118.2, *p* < 0.001; hM4D(Gi) ⨯ holding potential interaction, *F*_7,147_ = 3.1, *p* = 0.005; *n* = 11–12 cells from 4 rats). **E** I_h_ current density was significantly increased in hM4D(Gi)-expressing rats compared with mCherry-expressing rats (*t*-test, *t*_21_ = 2.1, *p* = 0.049, *n* = 11–12 cells). **F**, **G** Yoked DCZ infusions led to a significant depolarizing shift of the *V*_1/2_ in hM4D(Gi)-expressing rats compared to mCherry-expressing rats (*t*-test, *t*_21_ = 2.4, *p* = 0.023, *n* = 11–12 cells). **H** Representative temporal summation of evoked EPSPs (50 Hz ⨯ 5) recorded from mCherry and hM4D(Gi)-expressing VTA dopamine neurons in TH-Cre rats that received yoked DCZ infusions. **I** Compared with mCherry-expressing VTA dopamine neurons, hM4D(Gi)-expressing dopamine neurons exhibited a smaller increase in temporal summation of EPSPs. ZD7288 induced greater increase in temporal summation of EPSPs in hM4D(Gi)-expressing rats (two-way RM ANOVA, hM4D(Gi), *F*_1,11_ = 0.004, *p* = 0.856; ZD7288, *F*_1,11_ = 56.1, *p* < 0.001; hM4D(Gi) ⨯ ZD7288 interaction, *F*_1,11_ = 16.4, *p* = 0.002; *n* = 6–7 cells from 3 rats). **J**, **K** Representative Co-IP reaction was performed using anti-HCN3 (**J**) or HCN4 (**K**) antibodies in VTA lysate from rats that expressed mCherry and hM4D(Gi) in VTA dopamine neurons and received yoked DCZ administration. IP, immunoprecipitation; IB, immunoblotting. (HCN3: *t*-test, *t*_18_ = 2.2, *p* = 0.044, *n* = 9 and 9. HCN4: *t*-test, *t*_18_ = 2.3, *p* = 0.034, *n* = 9 and 9). For the entire figure, *p < 0.05, **p < 0.01, ***p < 0.001.

What might be the mechanism for the cocaine self-administration-induced differential modulation of I_h_ in dopamine and GABA neurons? D_2_ (*Drd2*) mRNA was predominantly expressed in dopamine neurons, raising the possibility that a chronic decrease in cAMP via cocaine-induced D_2_ receptor activation may underlie the I_h_ upregulation. Tetratricopeptide repeat-containing Rab8b-interacting protein (TRIP8b) is an auxiliary HCN channel subunit [47] responsible for HCN trafficking to the plasma membrane and enrichment at dendrites [44, 48]. TRIP8b competes with cAMP for binding of the CNBD of HCN channels, and chronic increased cAMP via Gs-DREADD stimulation was shown to disrupt TRIP8b binding and HCN surface trafficking in hippocampal CA1 pyramidal neurons [10]. Immunohistochemistry showed that TRIP8b was expressed in the VTA and colocalized with TH^+^ dopamine neurons (Fig. S14A). In the Allen Brain Atlas mouse in situ hybridization (ISH) database (https://mouse.brain-map.org), TRIP8b (also called Pex5l) is abundantly expressed in the VTA (Fig. S14B). One possibility is that the chronic decrease in cAMP during the 10-day cocaine self-administration or yoked cocaine infusions induced an enhanced TRIP8b binding with HCN channels and enhanced their surface trafficking, which provides a potential mechanism for the augmented I_h_ and dampened summation of EPSPs. To test the hypothesis, we investigated whether chronic Gi-DREADD stimulation via yoked delivery of deschloroclozapine dihydrochloride (DCZ) mimicked the effect of cocaine on I_h_ upregulation. DCZ is a highly brain-penetrant DREADD actuator that shows greater affinity and potency for DREADDs [49]. AAV8-hSyn-DIO-hM4D(Gi)-mCherry or the control vector (AAV8-hSyn-DIO-mCherry) was injected into the VTA of TH-Cre rats (Fig. 5B, left). Two weeks after AAV expression, we examined the expression the AAVs in midbrain sections (Fig. 5A). The AAVs, as indicated by mCherry, were expressed in 79.9 ± 4.2% of TH^+^ dopamine neurons (green) but were not expressed in TH^−^ neurons in the VTA (Fig. 5B, right). Pressure ejection of DCZ (1 µM) via a patch pipette resulted in hyperpolarization and a pause of AP firing in hM4D(Gi)-expressing VTA dopamine neurons but did not affect AP firing in mCherry-expressing neurons (Fig. S15).

Next, hM4D(Gi)- or mCherry-expressing rats received 10 days of i.v. DCZ administration yoked to the infusion schedule of rats that self-administered cocaine. VTA slices or tissue punches were obtained one day after the last DCZ administration for electrophysiology and coimmunoprecipitation, respectively. Compared with those which received the mCherry control, I_h_ amplitude and I_h_ density were significantly increased in hM4D(Gi)-expressing VTA dopamine neurons (Fig. 5C–E), and there was a depolarizing shift of *V*_1/2_ (Fig. 5F, G). hM4D(Gi)-expressing neurons exhibited dampened temporal summation of EPSPs (Fig. 5H, I), and the HCN blocker ZD7288 induced a greater increase in temporal summation of EPSPs in these neurons (Fig. 5H, I). Together, these results suggest chronic Gi-DREADD activation induces an upregulation of HCN function at somata and dendrites of VTA dopamine neurons. Finally, we investigated the mechanism that may underlie the upregulation of Ih induced by chronic hM4D(Gi) stimulation. We performed Co-IP using anti-HCN3 and -HCN4 antibodies as these HCNs are the main isoforms expressed in VTA dopamine neurons. We found that more TRIP8b bound to HCN3 or HCN4 in VTA tissue punches from rats that expressed hM4D(Gi) compared with rats that expressed the control vector mCherry following chronic yoked DCZ administration (Fig. 5J, K, Fig. S16). These results suggest that chronically decreased cAMP leads to increases in the TRIP8b-HCN interaction and surface trafficking of HCN channels.

### Effects of intra-VTA infusions of ivabradine on cocaine self-administration

**Fig. 6:**
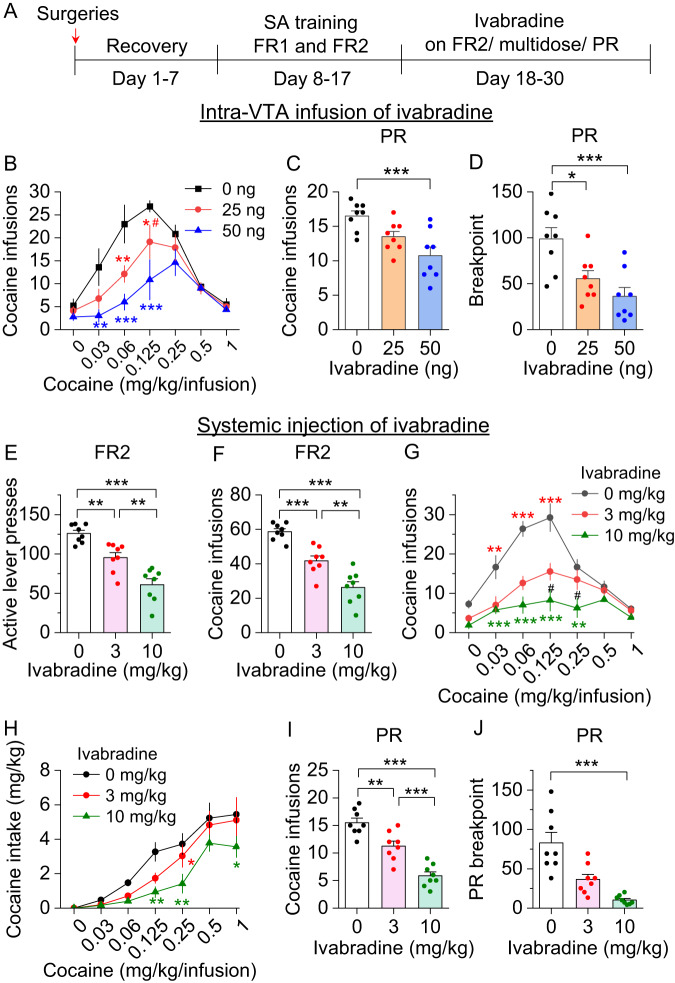
Ivabradine dose-dependently decreased cocaine intake under FR/PR schedules and produced a downward shift of the cocaine dose-response curve. **A** Timeline of catheterization, cocaine self-administration and ivabradine treatment. **B** Intra-VTA infusion of Ivabradine produced a significant downward shift in the number of cocaine infusions on the cocaine dose-response curve (two-way RM ANOVA: Ivabradine, *F*_2,21_ = 13.0, *p* < 0.001; cocaine dose, *F*_6,126_ = 22.0, *p* < 0.001; Ivabradine ⨯ cocaine dose interaction, *F*_6,126_ = 2.5, *p* = 0.005; 25 ng vs 0 ng, red *; 50 ng vs 0 ng, blue *; 25 ng vs 50 ng, red ^#^; *n* = 8 rats for each group). C Intra-VTA infusion of significantly attenuated the number of cocaine infusions under PR reinforcement conditions (one-way ANOVA, *F*_2,21_ = 9.1, *p* = 0.001, n = 8 rats in each group). **D** Intra-VTA infusion of significantly attenuated the PR breakpoint (one-way ANOVA, *F*_2,21_ = 9.7, *p* = 0.001, *n* = 8 rats in each group). **E**, **F** Systemic ivabradine administration (0, 3, 10 mg/kg, i.p.) following iv elacridar (5 mg/kg), dose-dependently decreased the mean number of active lever presses (**E**, one-way ANOVA: *F*_2,23_ = 27.3, *p* < 0.001) and cocaine infusions under an FR2 reinforcement schedule (**F**, one-way ANOVA: *F*_2,23_ = 35.6, *p* < 0.001) (*n* = 8 rats in each group). **G**, **H** Ivabradine pretreatment produced a significant downward shift in the dose-response curve for cocaine infusions (**G**, two-way RM ANOVA: ivabradine pretreatment, *F*_2,21_ = 16.9, *p* < 0.001; cocaine dose, *F*_6,126_ = 28.0, *p* < 0.001; ivabradine pretreatment × cocaine dose interaction, *F*_12,126_ = 6.3, *p* < 0.001; 3 vs. 0 mg/kg ivabradine, red *; 10 vs. 0 mg/kg ivabradine, green *; 3 vs. 10 mg/kg ivabradine black ^#^) and decreased total cocaine intake (H, two-way RM ANOVA: ivabradine pretreatment, *F*_2,21_ = 5.8, *p* = 0.010; cocaine dose, *F*_6,126_ = 68.7, *p* < 0.001; ivabradine × cocaine dose interaction, *F*_12,126_ = 1.6, *p* = 0.090; 10 vs. 0 mg/kg ivabradine green *; 3 vs. 10 mg/kg ivabradine red *) on the dose-response curve (*n* = 8 rats in each group). **I**, **J** Ivabradine dose-dependently decreased the number of cocaine infusions (**I**, one-way ANOVA: *F*_2,23_ = 34.2, *p* < 0.001; *n* = 8 rats in each group) and breakpoint (**J**, Brown-Forsythe equal variance: *p* < 0.05; Kruskal-Wallis one-way ANOVA on ranks: ivabradine, *H* = 17.6, *p* < 0.001; *n* = 8 rats in each group) under a PR reinforcement schedule. *p < 0.05, **p < 0.01, ***p < 0.001; ^#^*p* < 0.05, ^##^*p* < 0.01, ^###^*p* < 0.001.

Does the upregulation of HCN function in VTA dopamine neurons contribute to motivated drug taking? To determine this, we examined whether intra-VTA infusion of ivabradine, a clinically approved HCN blocker [21], affected cocaine self-administration in rats. Rats were trained to self-administer cocaine under a fixed ratio-1 schedule (FR1, 1 mg/kg/infusion) for 5 days, followed by a FR2 schedule (0.5 mg/kg/infusion) for another 5 days (Fig. 6A). We found that bilateral intra-VTA infusions of ivabradine (25 and 50 ng/0.5 µl/side) did not significantly affect cocaine self-administration under FR2 reinforcement (Fig. S17A–C). As 0.5 mg/kg/infusion cocaine lies on the descending limb of the cocaine dose-response curve [50–54], we next examined whether ivabradine alters cocaine self-administration across a full range of cocaine doses. After 10 days of cocaine self-administration training, rats that met criteria for stable cocaine self-administration were subsequently trained in a single self-administration session maintained by a full range of cocaine doses under a FR2 reinforcement schedule. We observed classic inverted U-shaped dose-response curves, and maximal infusions occurred at the 0.125 mg/kg/infusion dose (Fig. 6B). After rats achieved stable cocaine self-administration in this multiple dose paradigm, ivabradine (25 and 50 ng/0.5 µl/side) or vehicle was infused into the VTA 10 min prior to cocaine self-administration. Ivabradine led to dose-dependent downward shifts of the cocaine dose-response curve (Fig. 6B). We next examined whether intra-VTA ivabradine alters cocaine self-administration under a progressive ratio (PR) schedule of reinforcement [55]. Intra-VTA infusions of ivabradine dose-dependently reduced cocaine infusions (Fig. 6C) and breakpoints (Fig. 6D) compared to vehicle infusions, suggesting reduced motivation to obtain cocaine when the effort required is progressively increased. The location of cannula implantation was verified after the experiments (Fig. S18).

### Effects of systemic injections of ivabradine on cocaine self-administration

We next determined whether systemic HCN blockade also affects motivated drug taking. To test this, rats were trained to self-administer cocaine under FR1/FR2 for 10 days (1 and 0.5 mg/kg/infusion, Fig. 6A). On subsequent days, we examined the effects of systemic administration of ivabradine or vehicle on cocaine self-administration under a FR2 schedule with 0.5 mg/kg/infusion cocaine. Currently available HCN channel blockers, including ivabradine, show a very limited ability to cross the blood-brain barrier (BBB) due to p-glycoprotein-mediated efflux, unless delivered with the p-glycoprotein inhibitor elacridar [56]. Rats first received an i.v. injection of elacridar (5 mg/kg) followed by an i.p. injection of ivabradine (3 or 10 mg/kg) or vehicle (0 mg/kg) 10 min prior to cocaine self-administration. Ivabradine (with elacridar) dose-dependently reduced active lever presses (Fig. 6E) and cocaine infusions (Fig. 6F) compared to vehicle (with elacridar) but had no significant effect on inactive lever presses (Fig. S19).

We next examined the effects of ivabradine on the cocaine dose response curve in a separate cohort of rats. After acquiring stable cocaine self-administration in the multiple dose paradigm, rats received elacridar (5 mg/kg, i.v.) followed by ivabradine (3 or 10 mg/kg, i.p.) or vehicle (0 mg/kg) injection 10 min prior to multidose cocaine self-administration. Ivabradine pretreatments led to a dose-dependent downward shift in the cocaine dose-response curve and an attenuation of cocaine intake, the shift was more prominent on the ascending limb at the lower dose ivabradine (3 mg/kg; Fig. 6G, H). Finally, we determined whether ivabradine affects the motivation for cocaine self-administration under a PR reinforcement schedule. Elacridar and ivabradine treatments were performed as described above, and the effects of ivabradine (3 or 10 mg/kg, i.p.) or vehicle on cocaine infusions and breakpoint were examined. Ivabradine pretreatment dose-dependently reduced cocaine infusions (Fig. 6I) and breakpoint (Fig. 6J) compared to vehicle-treated rats. Thus, ivabradine reduced the motivation to obtain cocaine when the effort required is progressively increased. Taken together, the above results suggest that ivabradine reduces cocaine intake by decreasing the motivation for cocaine taking.

### Effects of systemic injections of ivabradine on oral sucrose self-administration

To determine whether ivabradine affects non-drug reinforcement, we investigated the effects of ivabradine on oral self-administration of sucrose pellets under both FR2 and PR schedules. Rats established stable oral sucrose self-administration during the 10-day training period which emulated the procedure for cocaine self-administration training. On subsequent days, the effects of ivabradine (0, 3 or 10 mg/kg, i.p.) in combination with i.v. injection of elacridar (5 mg/kg) on sucrose self-administration were tested. In contrast to cocaine self-administration, when tested for effects on sucrose self-administration under a FR2 schedule, ivabradine at 3 or 10 mg/kg had no significant effects on active and inactive lever presses or sucrose pellets earned (Fig. S20A–C) compared to vehicle and elacridar. Moreover, while ivabradine reduced sucrose pellet self-administration under the PR schedule, these effects were only observed at the high (i.e., 10 mg/kg) dose at which significant decreases in sucrose pellets earned and breakpoint were found (Fig. S20D, E). In contrast to cocaine self-administration, significant effects of the lower (i.e., 3 mg/kg) ivabradine dose were not observed (Fig. S20D, E). Altogether, these findings demonstrate that ivabradine selectively influences cocaine self-administration, with effects on motivation to obtain a natural reward observed only at a higher ivabradine dose and under conditions where the effort required to obtain the natural reward is greater.

## Discussion

Here, we show that both VTA dopamine and GABA neurons expressed cAMP-sensitive HCN mRNA (*Hcn2* and *Hcn4*) and that I_h_ in these neurons was sensitive to cAMP stimulation. Acute bath application of cocaine and chronic self-administration led to opposite changes in the voltage-dependent activation of I_h_ in VTA dopamine neurons without significantly altering I_h_ in VTA GABA neurons. As D_2_ (*Drd2*) mRNA was predominantly expressed in VTA dopamine neurons, cocaine-induced activation of D_2_ receptors and the resultant decrease in cAMP likely contributes to the I_h_ modulation. In support of this idea, chronic Gi-DREADD stimulation in VTA dopamine neurons mimicked the effect of cocaine self-administration on I_h_ upregulation. Finally, we demonstrated that intra-VTA or systemic injections of ivabradine reduced cocaine intake at low unitary cocaine doses and the motivation to obtain cocaine when effort demand is high. These results reveal a critical role of HCN channels in regulating motivated cocaine taking.

Major efforts have been directed towards the identification of VTA dopamine and GABA neurons based on electrophysiological criteria. The presence of I_h_ currents had historically been used as the key criterion for the identification of midbrain dopamine neurons in brain slices. Through a combination of whole-cell recordings, biocytin loading and *post hoc* immunohistochemistry with TH and GAD antibodies, Margolis et al. reported that both VTA dopamine neurons and GABA neurons exhibited I_h_ currents with comparable amplitude in rat brain slices [37, 40]. We confirmed these findings with TH-tdTomato fluorescent reporter rats and single-cell PCR that provided unequivocal cell type identification of recorded neurons. Immunohistochemistry has shown that HCN2 is expressed in the rat VTA [36] and that HCN4 is expressed in VTA dopamine and GABA neurons [37]. Although earlier whole-brain in situ hybridization analysis has shown that all *Hcn1–4* isoforms are expressed in the VTA [57], the specific cell types that express different HCN isoforms have not been identified, and their expression has not been directly compared between cell types. We utilized RNAscope to quantify the expression of *Hcn1–4* mRNA in defined VTA neuron populations. We found that VTA GABA neurons expressed all 4 *Hcn* mRNA isoforms (*Hcn1–4*), while VTA dopamine neurons expressed predominantly *Hcn3–4*. RNAscope allows detection of single mRNA transcripts with high specificity and sensitivity [57]. Our results provide a high-resolution, quantitative comparison of the specific HCN isoforms expressed in two major neuronal types of the VTA and overcomes the sampling size limitation inherent in whole cell recordings. Previous studies have shown that differences in the amplitude of I_h_ currents in VTA neurons depend on the projection targets [13, 58–61]. Future studies will be carried out to determine *Hcn1–4* mRNA expression in VTA neurons that project to different brain targets.

HCN channels are often assembled as heterotetramers in native neurons [1, 33, 34]. HCN2 and HCN4 are highly sensitive to cAMP, whereas HCN1 and HCN3 are relatively insensitive to cAMP [3, 38, 39]. Both RNAscope and electrophysiological recordings indicate that HCN channels expressed on VTA dopamine and GABA neurons are sensitive to cAMP. Indeed, increasing intracellular cAMP with forskolin and rolipram produced similar depolarizing shifts of *V*_1/2_ of I_h_ in VTA dopamine and GABA neurons. However, bath application of cocaine led to D_2_-dependent hyperpolarizing shifts of *V*_1/2_ in VTA dopamine neurons but did not alter I_h_ in VTA GABA neurons. D_2_ (*Drd2*) mRNA was predominantly expressed in VTA dopamine neurons, whereas D_1_ (*Drd1*) mRNA was barely detectable in the VTA, which may explain the lack of significant effects of cocaine on I_h_ in VTA GABA neurons.

Acute and chronic Gs-DREADD stimulation produced opposite modulation of HCN function in hippocampal CA1 pyramidal neurons and depressive-like behaviors via cAMP-dependent mechanisms [10]. However, whether this cAMP-induced regulation of HCN can be induced by animals’ behavioral experiences remained previously unknown. We found that acute bath application of cocaine and chronic cocaine self-administration produced opposite modulation of I_h_ currents in VTA dopamine neurons but did not alter I_h_ in VTA GABA neurons. Several lines of evidence suggest that a chronic decrease in cAMP via D_2_ receptor activation mediates the I_h_ modulation in the VTA dopamine neurons. First, *Drd2* mRNA is predominantly expressed in dopamine neurons, while cocaine alters I_h_ only in dopamine neurons. Second, we found that yoked cocaine infusions and chronic Gi-DREADD stimulation also led to an upregulation of I_h_ currents in VTA dopamine neurons. Thus, all three manipulations that induced a chronic decrease in cAMP in dopamine neurons caused a similar upregulation of I_h_ currents and reduced temporal summation of EPSPs, suggesting a common mechanism. However, it is important to acknowledge that genetically engineered receptors are unlikely to fully replicate the complex mechanisms of endogenous receptor activation.

The auxiliary subunit TRIP8b is required for the trafficking of HCN channels to the membrane surface and enrichment at dendrites [44, 48]. TRIP8b competes with cAMP for binding of HCN channels via an allosteric inhibitory mechanism [62–64]. Chronic Gs-DREADD stimulation in hippocampal CA1 pyramidal neurons impaired HCN cell surface trafficking via disruption of TRIP8b-HCN interactions [10]. As TRIP8b is highly enriched in midbrain dopamine neurons (Fig. S14), it is likely that chronically decreased cAMP causes increased TRIP8b-HCN interaction, resulting in increased HCN channel cell surface trafficking. In support of this idea, we have shown that chronic hM4D(Gi) stimulation led to an increase in TRIP8b binding to HCN3 and HCN4 in the VTA. Increased HCN trafficking to the cell membrane and dendrites of VTA dopamine neurons likely underlies the observed decrease in temporal summation of EPSPs via decreased input resistance.

Our findings that cocaine self-administration induced an upregulation of I_h_ currents stand in contrast to an earlier study showing that 7 days of cocaine (15 mg/kg) i.p. injections in rats decreased I_h_ amplitude in putative VTA dopamine neurons by ~40%, which was accompanied by a reduction in cell capacitance of similar magnitude (~33%), leaving I_h_ density unaltered [65]. Although we cannot exclude the possibility that i.p. cocaine injections and i.v. cocaine administration produced differential modulation of I_h_ currents in VTA dopamine neurons, other factors may contribute to the differences. Of note, VTA neurons in that study were identified as dopaminergic only by the presence of I_h_ currents, and the minimum threshold value for I_h_ amplitude was not defined [65]. Studying changes in I_h_ currents in dopamine neurons while concurrently using I_h_ as a marker for neuron identification may introduce potential bias and sampling error. Indeed, the averaged I_h_ current amplitude in the saline group was ~500 pA at −130 mV [65], which was considerably larger than the I_h_ currents in our saline group and those previously reported in drug-naïve rats [40]. Additionally, i.p. injection of cocaine for 7 days leads to a 33% decrease in membrane capacitance [65]. As there is a linear relationship between cell capacitance and membrane surface area [66], the decrease in cell capacitance was interpreted as a decrease in soma size of VTA dopamine neurons [65]. However, both the present and a prior study [67] found that cocaine self-administration did not significantly change the soma size of VTA dopamine neurons as determined by TH immunofluorescence as well as cell capacitance.

HCN channels are abundantly expressed in the VTA, yet it was previously unknown whether HCN channels contribute to motivated drug taking behavior. As chronic cocaine self-administration upregulated I_h_ currents in VTA dopamine neurons, we tested whether a clinically approved HCN blocker could reduce cocaine self-administration. Ivabradine is a nonselective blocker of all 4 HCN isoforms [68, 69] and does not produce significant off-target effects on voltage-gated Na^+^, Ca^2+^, and K^+^ channels at concentrations that block HCN channels [70, 71]. We found that intra-VTA infusion of ivabradine produced a dose-dependent suppression of cocaine intake on a PR schedule and a downward shift of the cocaine dose-response curve. The decrease in operant responding on the ascending limb could be attributable to a decrease in the reinforcing efficacy of cocaine [72–74]. Cocaine-induced increases in dopamine levels in the striatum contribute to its reinforcing effects [16, 75]. Dopamine release is triggered by AP firing in dopamine neurons. HCN blockers significantly decreased spontaneous AP firing in these neurons [13–15]. HCN blockade is expected to decrease dopamine release in the NAc, which may explain the common decrease in operant responding when cocaine unitary doses were relatively low or when the effort required to obtain cocaine reward was progressively increased under PR reinforcement. However, intra-VTA infusion of ivabradine did not significantly affect the FR2 reinforcement schedule at 0.5 mg/kg/infusion and the descending limb of the inverted U-shaped dose-response curve, suggesting that higher unitary doses of cocaine remain similarly reinforcing despite HCN blockade. Ivabradine blocks HCN channels in the VTA non-discriminately. It is possible that HCN blockade in non-dopamine neurons may also contribute to the effects of ivabradine on cocaine intake.

Systemic injection of ivabradine produced similar effects on cocaine self-administration compared with intra-VTA ivabradine while producing an additional suppression of cocaine intake under a FR2 schedule. Ivabradine produced a downward, but not a rightward, shift of the cocaine dose response curve, suggesting that the reduction of the reinforcing strength of cocaine by ivabradine may not be compensated by increasing the amount of drug used per occasion. This feature is advantageous from a therapeutic perspective, as one cannot overcome the effect by taking greater cocaine doses [73]. Ivabradine is clinically approved for the treatment of heart failure [20, 21], and heart failure secondary to dilated cardiomyopathy is the leading cause of death in long-term cocaine users [19]. Cocaine increases heart rate and blood pressure via inhibition of catecholamine reuptake [76], while ivabradine is well tolerated and causes minimal side effects such as bradycardia [77]. Brain penetrant isoform-specific HCN blockers have also been developed and were shown to protect against seizures in mice [36]. Small-molecule inhibitors of the TRIP8b-HCN interaction may provide more selective targeting of neuronal HCNs [78]. There is therefore an opportunity to target HCNs for the treatment of cocaine use disorder and other mental disorders such as depression.

### Supplementary information

Supplementary methods and materials

Supplementary Figures 1-20

Source data for figures and supplementary figures

## Data Availability

All data supporting the findings of this study are documented within the paper and Supplementary materials, additional data are available from the corresponding author upon reasonable request. We have provided Source Data in Supplementary materials and statistical conclusion in figure legends.
