# Supplementary material for: cAMP-mediated upregulation of HCN channels in VTA dopamine neurons promotes cocaine reinforcement
Source: Mol Psychiatry. 2023 Oct 16;28(9):3930–42. doi: 10.1038/s41380-023-02290-x (PMC10730389; doi:10.1038/s41380-023-02290-x)
Supplement: Supplementary file 1 — Supplementary methods and materials [file 41380_2023_2290_MOESM1_ESM.docx]

**Supplemental materials and methods**

*Animals*

Long-Evans rats were purchased from Envigo (Indianapolis, IN). Heterozygous LE-Tg (TH-Cre)3.1Deis rats (RRRC: #00659) [1] and homozygous LE-Rosa26^em1(CAG-LSL-TdTomato)Rrrc^ (RRRC: #00938) reporter rats were obtained from the Rat Resource & Research Center (Columbia, MO). TH-Cre rats were crossed with Cre-dependent tdTomato reporter rats to produce TH-tdTomato reporter rats used for slice electrophysiology experiments. All the rat lines were maintained on the same Long-Evans background and were 7-9 weeks old at the beginning of the experiments. Roughly equal number of male and female rats were used for all experiments. All animals were housed in a room with regulated humidity (40-60%) and temperature (23 ± 1°C) and free access to water and food. Prior to experiments, rats were handled daily for 3-6 days. All animal maintenance and use were in accordance with protocols approved by the Institutional Animal Care and Use Committee of the Medical College of Wisconsin.

*RNAscope* in situ *hybridization*

After anesthesia by isoflurane inhalation, rats were euthanized by transcardial perfusion with 0.1 M sodium phosphate buffered saline (PBS) followed by 4% paraformaldehyde in PBS for 10-15 min. Brains were then rapidly frozen on dry ice. Following fixation, coronal midbrain sections (15 μm) were cut on a cryostat (Leica CM1860, Nussloch, Germany) and mounted on Superfrost Plus microscope slides (Fischer Scientific, Inc., Hampton, NH). Probes targeting *Rattus norvegicus* mRNA transcripts were designed by and purchased from Advanced Cell Diagnostics Inc (Hayward, CA), including *Slc6a3* (target region: NM_012694.2; base pairs (bp) 827-1913), *Gad1* (target region: NM_017007.1; bp 950-1872), *Hcn1* (target region: NM_053375.1; bp 1693-2793), *Hcn2* (target region: NM_053684.1; bp 196-2555), *Hcn3* (target region: *Hcn3 variant X5*, XM_039101560.1; bp 175-1948), *Hcn4* (target region: NM_021658.1; bp 1269-3852), *Drd1* (target region: NM_012546.2; bp 104-1053) and *Drd2* (target region: NM_012547.1; bp 445-1531). The experiment was carried out per the instructions provided by the manufacturer for the RNAscope Multiplex Fluorescent V2 Assay. Slides were mounted with Vectashield Antifade Mounting Medium (Vector Laboratories, Inc., Newark, CA). A Leica TCS SP8 confocal microscope was used to image the fluorescent probes. As a positive control, probes targeting the ubiquitously-expressed *Polr2a*, *Ppib*, and *Ubc* were used; as a negative control, a probe targeting the exogenous bacterial protein *Dapb* was used. Tyramide signal amplification (TSA®)-conjugated fluorescein, cyanine 3, and cyanine 5 (Akoya Biosciences, Marlborough, MA) were diluted to 1:1500. Identical experimental and confocal imaging settings were used to quantify and compare relative expression levels of mRNA that encode DAT, Gad1, HCN1, HCN2, HCN3, HCN4, D_1_ and D_2_ in VTA neuron populations, and imaging data was quantified using Imaris (Bitplane, Zürich, Switzerland). To quantify mRNA expression, the “spots” tool was used to identify and quantify individual fluorescent puncta, which represent fluorescently-labelled mRNA molecules [2]. Cyanine 5 was included to minimize nonspecific autofluorescence for the *Hcn1*, *Hcn2*, *Hcn3*, *Hcn4*, *Drd1* and *Drd2* probes. Negative control-stained slides were imaged using the same settings used for target probe imaging; this did not result in a notable signal, particularly for cyanine 5-linked targets.

*Immunohistochemistry*

After anesthesia by isoflurane inhalation, rats were euthanized by transcardial perfusion with 0.1 M sodium phosphate buffered saline (PBS) followed by 4% paraformaldehyde in PBS for 10-15 min. After perfusion, the brain was removed and post-fixed in the same fixative for 4 hours at 4°C and was then dehydrated in increasing concentrations of sucrose (20% and 30%) in 0.1 M PBS at 4°C and frozen on dry ice. After fixation, coronal VTA sections (20 µm) were cut with a Leica cryostat (CM1860). VTA sections were incubated with primary antibody against TRIP8b (Mouse monoclonal, 1:500, N212/17, BioLgend, San Deigo, CA) and/or tyrosine hydroxylase (TH, rabbit polyclonal, 1:300, Santa Cruz Biotechnology, Inc, Dallas, TX) at 4°C for 48 hr. After rinsing with PBS three times at 15 min each, VTA sections were then incubated in secondary antibodies: Goat anti-mouse IgG Alexa Fluor-488 and Goat anti-rabbit IgG Alexa Fluor-555 or Goat anti-rabbit Alexa Fluor-488 for 4 hours at room temperature in the dark. Confocal imaging was performed using a Leica SP8 upright confocal microscope.

*Slice preparation and electrophysiology*

Rats were anesthetized by isoflurane inhalation and perfused through the aorta with NMDG-based solution. The brain was removed, trimmed, and embedded in low-gelling-point agarose, and horizontal slices (200 μm thick) containing the VTA were cut using a vibrating slicer (Leica VT1200s, Nussloch, Germany), as described [3, 4]. The brain was sliced in the same NMDG-based solution containing (in mM): 92 NMDG, 2.5 KCl, 1.25 NaH_2_PO_4_, 0.5 CaCl_2_, 7 MgSO_4_, 30 NaHCO_3_, 25 glucose, 20 HEPES, 5 sodium ascorbate, 2 thiourea, and 3 sodium pyruvate. The VTA slices were cut at the midline to produce two individual slices, one from each hemisphere. After slice cutting, Na^+^ was gradually reintroduced into the NMDG solution by adding ACSF every 5 min for 20 min [3, 4]. The slices were allowed to rest in ACSF (in mM): 119 NaCl, 2.5 KCl, 2.5 CaCl_2_, 1 MgCl_2_, 1.25 NaH_2_PO_4_, 24 NaHCO_3_, and 10 glucose for at least an additional 30 min before transferring into the recording chamber. Solutions were saturated with 95% O_2_ and 5% CO_2_.

Whole-cell and cell-attached patch-clamp recordings were performed with patch-clamp amplifiers (Multiclamp 700B; Molecular Devices, San Jose, CA) and BX51WI infrared differential interference contrast (DIC) microscopy (Olympus). Data acquisition was performed using DigiData 1440A and 1550B digitizers and pClamp 10.7 (Molecular Devices). Signals were sampled at 10 kHz and filtered at 2 kHz. I_h_ currents, I_h_ activation curves, I_h_ tail current amplitudes, resting membrane conductance, membrane capacitance, and I_h_ density were measured as we have described previously [5, 6].To measure I_h_ current, 3 s hyperpolarizing steps from -60 to -130 mV were induced at -10 mV steps. Junction potentials between the pipette and bath ACSF were nullified prior to obtaining a seal. For I_h_ activation curves, 3 s hyperpolarizing steps to various potentials (-60 to -130 mV) were applied from a holding potential of -60 mV and tail currents were measured at -130 mV in the presence of tetraethylammonium chloride (TEA-Cl, 10 mM); the current following no hyperpolarizing step was measured and subtracted form tail current amplitudes at -130 mV, and the resulting values were plotted as a function of test potentials. The activation curve was fitted with a Boltzmann function, *I = I_max_/exp((V_m_ − V_1/2_)/s),* where the maximal tail current amplitude is *I_max_*, the test potential is *V*, the half-activation potential is *V_1/2_*, and the slope factor is *s*. Clampex software (Molecular Devices) was used to measure membrane capacitance by applying small amplitude (± 5 mV) hyperpolarizing and depolarizing steps. I_h_ density was determined by normalizing I_h_ amplitude at -130 mV to the cell capacitance. To measure temporal summation of EPSPs, a tungsten bipolar electrode was placed into the VTA at about 100 μm of the patched neuron. EPSPs were evoked by a train of electrical stimulation (50 Hz x 5) of excitatory synaptic inputs in the presence of GABA_A_ (picrotoxin, 50 µM), GABA_B_ (CGP55845, 1 µM), D_2_ (sulpiride, 1 µM) and NMDA (D-AP5, 50 µM) receptor antagonists. Temporal summation of EPSPs was expressed as an increase in synaptic depolarization occurring during a train and was calculated by dividing EPSP5 and EPSC1 (EPSC5/EPSC1). The glass recording pipettes (3-5 MΩ) were filled with internal solution containing (in mM): 140 K-gluconate, 10 KCl, 10 HEPES, 0.2 EGTA, 2 MgCl_2_, 4 Mg-ATP, 0.3 Na_2_GTP (pH 7.2 with KOH). All recordings were performed at 32 ± 1°C maintained by an automatic temperature controller (Warner Instruments LLC, Hamden, CT). Series resistance was monitored (10-20 MΩ) and data were discarded if the resistance changed by more than 20% during an experiment. For single-cell RT-PCR, all equipment was cleaned with RNase Zap. The glass recording pipettes were baked at 400 °C for at least 4 hours to deactivate RNase prior to use. Following completion of recording, gentle negative pressure was applied to aspirate cytoplasmic contents into the glass pipette while maintaining a Gigaohm seal. The pipette was then withdrawn slowly from the cell and positive pressure was used to dispense the collected contents into individual RNase-free microcentrifuge tubes for RT-PCR.

*Single-cell RT-PCR*

Complementary DNA (cDNA) was synthesized from the cytoplasmic mRNA using iScript™ Advanced cDNA Synthesis Kit (Bio-Rad Laboratories, Hercules, CA). Target genes were then amplified with two PCR steps using the following primer pairs. *Gapdh*, forward 5’- GCCATCAACGACCCCTTCAT-3’, reverse 5’- TTCACACCCATCACAAACAT-3’, 315 bp; *Th*, forward 5’- GTCACGTCCCCAAGGTTCAT-3’, reverse 5’- AGCATTCCCATCCCTCTCCT-3’, 159 bp; *Gad2*, forward 5’- TCTTTTCTCCTGGTGGTGCC-3’, reverse 5’- CCCCAAGCAGCATCCACAT-3’, 391 bp. Each PCR amplification consisted of 35 cycles, 94°C for 30 s, 58°C for 45 s, and 72°C for 1 min. The products of the second PCR were analyzed in 1.5 % agarose gels using ethidium bromide.

*Jugular catheterization surgery*

Rats were anesthetized with ketamine (90 mg/kg, i.p.) and xylazine (10 mg/kg, i.p.), and round-tip polyurethane catheter with a bead 3.8 cm from the catheter tip (C30PU-RJV1611, Instech Laboratories, Inc, Plymouth Meeting, PA) was inserted into the right jugular vein. The catheter was connected to a vascular access button (22-gauge; VABR1B/22, Instech) and implanted subcutaneously on the back of the rats. If required, stereotaxic surgery was performed immediately following jugular vein catheterization. After surgery, animals received a single subcutaneous injection of buprenorphine-SR (1 mg/kg), an analgesic. Catheters were flushed with 0.2 ml of heparinized saline (30 units/ml) and cefazolin (100 mg/ml). Rats were allowed to recover for about one week (~2 weeks for DREADD experiments) prior to the start of self-administration experiments. Catheter patency was tested by i.v. infusion of 0.05 ml xylazine (20 mg/ml) every week after cocaine self-administration or when compromise of catheter patency was suspected [7].

*The cAMP assay*

cAMP levels were measured with the cAMP ELISA kit (Enzo Life Sciences) according to the manufacturer’s protocol. Briefly, bilateral VTA was punched out and frozen in liquid nitrogen immediately following cocaine self-administration or yoked saline and cocaine administration. After collecting all tissue punches, VTA tissues from each rat were homogenized individually in ice-cold 0.1 M HCl. The homogenates were centrifuged at 13, 000 × *g* (5430R, Eppendorf, Enfield, CT) for 10 min at 4 °C to pellet debris. The supernatants were collected for ELISA assay. Absorbance was read at 405 nm with an ELX800 Universal Microplate Reader (Bio-TEK Instruments). Finally, the cAMP level was normalized to the total protein concentration, which was assayed using a BCA method.

*Co-Immunoprecipitation (Co-IP) reactions and western blotting*

Co-IP experiments were conducted following the manufacturer's instructions, using a commercial kit (#26149, ThermoFisher, Rockford, IL). First, rabbit anti-HCN3 (APC-057) or rabbit anti-HCN4 antibodies (APC-052, both from Alomone Labs, Jerusalem, Israel) were conjugated to AminoLink Plus Coupling Resin through 90–120-minute incubation in a coupling buffer at room temperature while gently end-over-end rotating. VTA tissue punches were homogenized in ice-cold IP lysis/wash buffer followed by sonication and centrifugation at 13000 x *g* (5430R) for 10 min at 4°C. Supernatant was transferred to a new tube and pre-cleared by incubation with control agarose resin at 4°C for 30 min. First, 50 μl of VTA lysate was set aside as an input fraction. Then, Co-IP was carried out by incubating 400 μl of remaining VTA lysate and pre-prepared HCN3 or HCN4 antibody-coupled resins on a rotator overnight at 4°C. The resins were washed with ice cold lysis/wash buffer, then eluted into 60 μl elution buffer followed by sample buffer dilution and boiling. Then 15 well polyacrylamide gels (10% for HCN3 and 7.5% for HCN4) with 10 μl of input and 15 μl of elution from Co-IP reactions were used for western blotting quantification. PVDF membrane was incubated with primary antibodies anti-HCN3 (1:200) or anti-HCN4 (1:200) and mouse anti-TRIP8b (1:1000, N212/17, BioLegend, San Diego, CA) overnight on a shaker at 4°C. For chemiluminescence detection, secondary antibodies were purchased from Bio-Rad and ECL substrate was purchased from ThermoFisher. Western blots were exposed on ImageQuant LAS4000 imaging system and analyzed using ImageQuant TL analysis software.

*Stereotaxic surgeries and AAV injections*

Rats underwent stereotaxic surgery to implant cannulas for intra-VTA infusions of a HCN blocker or inject AAVs into the VTA. Rats were anesthetized with ketamine (90 mg/kg, i.p.) and xylazine (10 mg/kg, i.p.) and placed into a Neurostar stereotaxic system (Neurostar, Tübingen, Germany). For intra-VTA microinjection experiments, guide cannulae (26 gauge; P1 Technologies, Roanoke, VA) were implanted 2.8 mm above the VTA at stereotaxic coordinates: AP, -5.3 mm; ML ± 2.4 mm; DV, −7.8 mm; 10° angle [8]. For DREADD experiments, a glass capillary Nanoinjector (Neurostar) or a Nanoject III Programmable Nano-liter Injector (Drummond Scientific Company, Broomall, PA) was used to inject AAV8-hSyn-DIO-hM4D(Gi)-mCherry or AAV8-hSyn-DIO-mCherry (200 nl each; Addgene, Watertown, MA) into the VTA of TH-Cre rats at stereotaxic coordinates: AP, -5.6 mm; ML ± 2.4 mm; DV, −7.8 mm; 10°, with a rate of 60 nl/min. The injector was kept in place for at least 5 min to ensure AAV diffusion from the injector tip (~30 μm). Rats were kept on a heating pad during surgery and after surgery for 2-3 hours to maintain adequate body temperature. Following surgeries, rats received a subcutaneous analgesic injection (buprenorphine-SR, 1 mg/kg).

*Fixed-ratio cocaine self-administration training and yoked administration*

Cocaine self-administration was conducted similarly to our previously published studies [3]. Rats with no prior lever training were placed into operant conditioning chambers (Med Associates Inc., Fairfax, VT) with an active and inactive lever in counterbalanced positions on one wall. Rats were allowed to self-administer cocaine for 10 days in 3-hour sessions. Active responses resulted in a cocaine infusion and illumination of a cue light above the active lever for 5 s, followed by a 10 s timeout during which active responses were recorded but did not result in further infusions. Inactive lever presses had no programmed consequence. During the first 5 days, one active response was required for one cocaine infusion (FR1, 1 mg/kg/infusion; 40-50 μl over 2.3-2.8 s based on body weight), during the last 5 days two responses were required for one infusion (FR2, 0.5 mg/kg/infusion). Rats that failed to fully acquire stable self-administration (≥ 25 infusions; >2:1 active/inactive response ratio for 3 consecutive test sessions; < 20% variation in daily drug infusions across 2 consecutive test sessions) after 10 days were excluded from further study. Timepoints of cocaine infusions from rats that successfully acquired stable self-administration were used to yoke the i.v. delivery of saline, cocaine (FR1, 1 mg/kg/infusion; FR2, 0.5 mg/kg/infusion), and DCZ (FR1, 4 µg/kg/infusion; FR2, 2 µg/kg/infusion) in additional cohorts of rats. One day following self-administration or yoked administration, slices were prepared for patch-clamp electrophysiology, as described above.

*Examination of the effects of intra-VTA infusions or systemic injections of ivabradine on FR2 cocaine self-administration*

Rats underwent stereotaxic surgery, jugular catheterization, and 10 days of cocaine self-administration training under FR1/FR2 reinforcement, as described above (Fig. 6A). To examine the effects of intra-VTA infusions of ivabradine on FR2 cocaine self-administration, the day following completion of training, vehicle (0.5 μl/side) or ivabradine (25 or 50 ng/0.5 µl/side) were bilaterally microinjected into the VTA at a rate of 0.5 μl/min for 2 min via injector cannulae (33-gauge; P1 Technologies, Roanoke, VA) that were placed inside the previously implanted guide cannulae. The injector cannulae were connected through C313C connectors to 2 μl-Hamilton micro-syringes. The injectors were kept in place for an additional 2 min to ensure adequate drug diffusion from the injector tip. Cocaine self-administration under FR2 reinforcement was tested 10 min later. To examine the effects of systemic injections of ivabradine on FR2 cocaine self-administration, the day following completion of training, rats first received i.v. injection of elacridar (5 mg/kg) followed by i.p. injection of ivabradine (3 or 10 mg/kg) or vehicle (0 mg/kg). Cocaine self-administration under FR2 reinforcement was tested 10 min later.

*Examination of the effects of intra-VTA microinjection or systemic injections of ivabradine on multiple-dose cocaine self-administration.*

Rats received stereotaxic surgery, jugular catheterization, and 10 days of cocaine self-administration training under FR1/FR2 reinforcement, as described above. Rats that acquired stable cocaine self-administration were next trained to self-administer cocaine maintained by a full range of doses in a single session under FR2 reinforcement. Training sessions under the multiple-dose schedule began with 30 min at the cocaine self-administration training dose (0.5 mg/kg/infusion, 5.77 s duration), then another six 30 min trials in which different doses of cocaine were presented in a descending order [9] (1.0, 0.25, 0.125, 0.0625, 0.03125, and 0.0 mg/kg/infusion; infusion durations were 11.54, 2.88, 1.44, 0.72, 0.36, 0 s, respectively), each dose trial was separated by a 1-min intertrial timeout period. Stable responding was defined as < 20% variation in total cocaine infusions for two consecutive test sessions and the cocaine dose that maintained maximal response rates varied by no more than one-half log unit over two consecutive test sessions [10, 11]. When stable responding was attained, the effects of intra-VTA microinjections of HCN blockers (0, 25 or 50 ng/0.5 µl/side), intravenous elacridar (5 mg/kg) in combination with intraperitoneal ivabradine (0, 3 or 10 mg/kg) were examined, as described above.

*Examination of the effects of intra-VTA infusions or systemic injections of ivabradine on progressive-ratio cocaine self-administration.*

Another cohort of rats that had attained stable self-administration during the 10-day training period was trained to self-administer cocaine under a progressive-ratio schedule of reinforcement. Under a PR schedule, each cocaine infusion requires a progressively larger number of operant responses according to the equation *Ν* = 5 (*e^infusion × 0.18^* -1) [12] until a breakpoint (the number of lever presses completed for the last cocaine infusion prior to a 1-hour period during in which no infusions were obtained) is reached. After PR breakpoint stabilized (< 15% variability in number of infusions) for 3 consecutive test sessions, the effects of intra-VTA infusions of ivabradine, intraperitoneal injection of ivabradine or subcutaneous DCZ were examined, as described above.

*Examination of the effects of systemic injections of ivabradine on oral sucrose self-administration*

The procedures for oral sucrose self-administration testing were identical to the procedure used for cocaine self-administration [3, 13], except that active lever press led to delivery of a sucrose pellet. The method has been detailed in our recent publications [3, 13].

*Chemical reagents*

ZD7288, (RS)-(±)-sulpiride, rolipram, forskolin and ivabradine hydrochloride were purchased from Tocris Bioscience (Ellisville, MO). DCZ dihydrochloride (water soluble) was purchased from Hello Bio Inc. (Princeton, NJ). Elacridar hydrochloride was purchased from Medchemexpress LLC (Monmouth Junction, NJ). Cocaine HCl was provided by the NIDA Drug Supply Program. All other common chemicals were obtained from Sigma-Aldrich (St. Louis, MO). Ivabradine, DCZ and cocaine HCl were dissolved in sterile saline (Midwest Veterinary Supply, Sun Prairie, WI), diluted to unit doses appropriate for behavioral studies, and filtered through a 0.2 μm membrane prior to use. Elacridar was first dissolved in DMSO to make 62.5 mg/ml stock solution, aliquoted and kept in a –80°C freezer. Before use, the stock solution was first mixed with PEG300 and then diluted with saline (0.9% NaCl) to obtain a working solution (DMSO:PEG300:saline = 1:4:15 solution). The working solution was filtered through a 0.2 μm membrane prior to use.

*Statistics*

Data are presented as the mean ± SEM. Data sets were compared with either Student’s *t*-test, Paired *t*-test, one-way ANOVA followed by Tukey’s *post hoc* analysis, or two-way repeated measures (RM) ANOVA. *Post hoc* analyses were performed only when ANOVA yielded a significant main effect or a significant interaction between the two factors. As the analysis of breakpoints for the progressive ratio violates the assumption of homogeneity of variance [12], we used Kruskal-Wallis one-way ANOVA on ranks followed by Dunn's *post hoc* analysis for pair-wise comparisons. The cumulative distributions for neuronal surface area from yoked-saline or cocaine self-administration rats were compared with Kolmogorov-Smirnov test (K-S test). Results were considered to be significant at *p* < 0.05.

**References**

1. Witten IB, Steinberg EE, Lee SY, Davidson TJ, Zalocusky KA, Brodsky M *et al.* Recombinase-driver rat lines: tools, techniques, and optogenetic application to dopamine-mediated reinforcement. *Neuron* 2011; **72**(5)**:** 721-733.

2. Wang F, Flanagan J, Su N, Wang LC, Bui S, Nielson A *et al.* RNAscope: a novel in situ RNA analysis platform for formalin-fixed, paraffin-embedded tissues. *The Journal of molecular diagnostics : JMD* 2012; **14**(1)**:** 22-29.

3. Mu L, Liu X, Yu H, Hu M, Friedman V, Kelly TJ *et al.* Ibudilast attenuates cocaine self-administration and prime- and cue-induced reinstatement of cocaine seeking in rats. *Neuropharmacology* 2021; **201:** 108830.

4. Ting JT, Lee BR, Chong P, Soler-Llavina G, Cobbs C, Koch C *et al.* Preparation of Acute Brain Slices Using an Optimized N-Methyl-D-glucamine Protective Recovery Method. *Journal of visualized experiments : JoVE* 2018; (132).

5. Zhong P, Vickstrom CR, Liu X, Hu Y, Yu L, Yu HG *et al.* HCN2 channels in the ventral tegmental area regulate behavioral responses to chronic stress. *Elife* 2018; **7:** doi: 10.7554/eLife.32420.

6. Yu H, Liu X, Chen B, Vickstrom CR, Friedman V, Kelly TJ *et al.* The Neuroprotective Effects of the CB2 Agonist GW842166x in the 6-OHDA Mouse Model of Parkinson's Disease. *Cells* 2021; **10**(12).

7. Wright KN, Strong CE, Addonizio MN, Brownstein NC, Kabbaj M. Reinforcing properties of an intermittent, low dose of ketamine in rats: effects of sex and cycle. *Psychopharmacology* 2017; **234**(3)**:** 393-401.

8. Paxinos G, Watson C. *The Rat Brain in Stereotaxic Coordinates, 6th Edition*. Academic Press2006, 456pp.

9. Anderson EM, Larson EB, Guzman D, Wissman AM, Neve RL, Nestler EJ *et al.* Overexpression of the Histone Dimethyltransferase G9a in Nucleus Accumbens Shell Increases Cocaine Self-Administration, Stress-Induced Reinstatement, and Anxiety. *J Neurosci* 2018; **38**(4)**:** 803-813.

10. Xi ZX, Song R, Li X, Lu GY, Peng XQ, He Y *et al.* CTDP-32476: A Promising Agonist Therapy for Treatment of Cocaine Addiction. *Neuropsychopharmacology* 2017; **42**(3)**:** 682-694.

11. Keck TM, Zou MF, Bi GH, Zhang HY, Wang XF, Yang HJ *et al.* A novel mGluR5 antagonist, MFZ 10-7, inhibits cocaine-taking and cocaine-seeking behavior in rats. *Addiction biology* 2014; **19**(2)**:** 195-209.

12. Richardson NR, Roberts DC. Progressive ratio schedules in drug self-administration studies in rats: a method to evaluate reinforcing efficacy. *Journal of neuroscience methods* 1996; **66**(1)**:** 1-11.

13. Liu X, Vickstrom CR, Yu H, Liu S, Snarrenberg ST, Friedman V *et al.* Epac2 in midbrain dopamine neurons contributes to cocaine reinforcement via enhancement of dopamine release. *Elife* 2022; **11**.
