# Supplementary material for: cAMP-mediated upregulation of HCN channels in VTA dopamine neurons promotes cocaine reinforcement
Source: Mol Psychiatry. 2023 Oct 16;28(9):3930–42. doi: 10.1038/s41380-023-02290-x (PMC10730389; doi:10.1038/s41380-023-02290-x)
Supplement: Supplementary file 2 — Supplementary Figures 1-20 [file 41380_2023_2290_MOESM2_ESM.pdf]

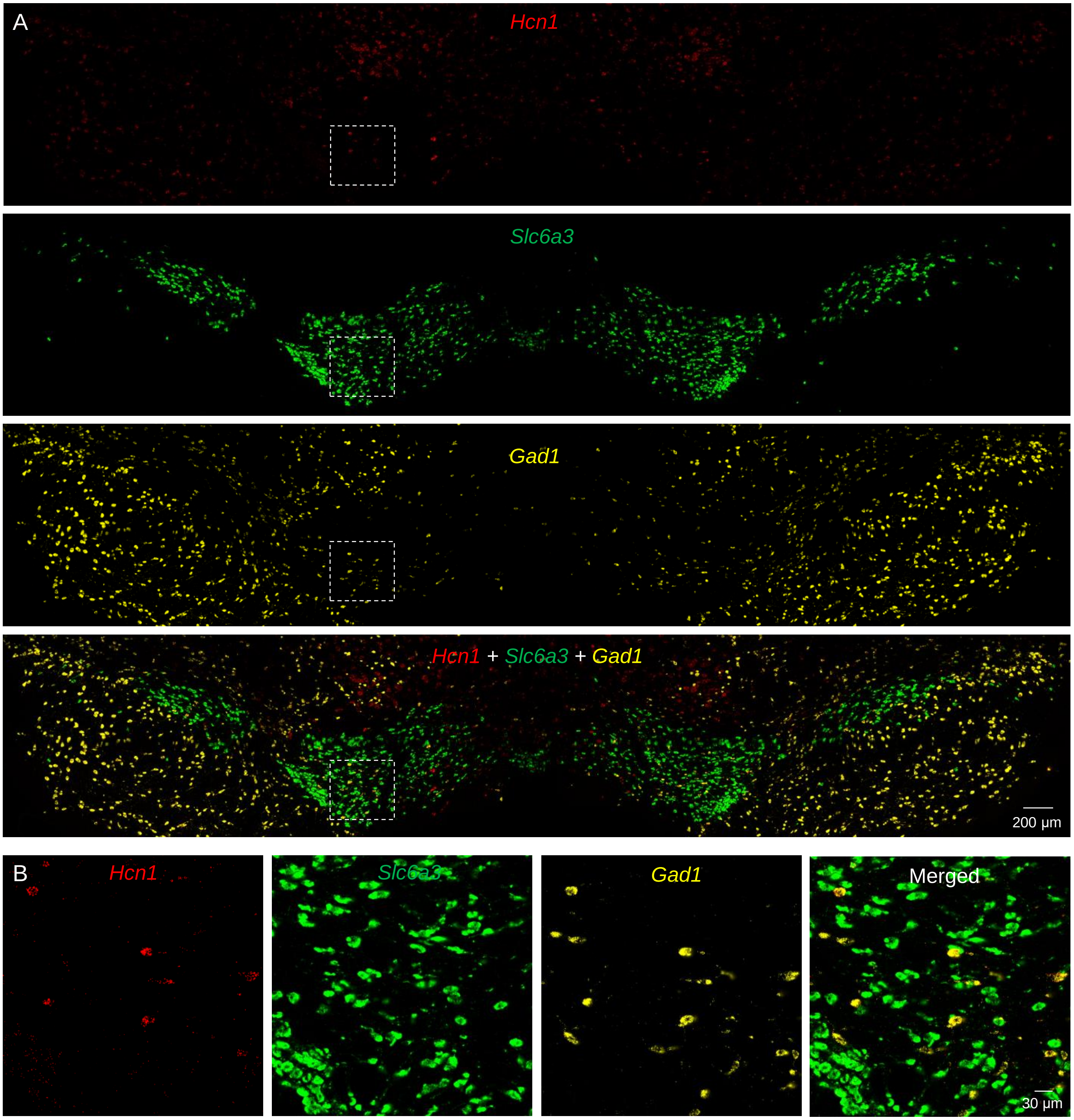

Figure S1. Expression of *Hcn1* mRNA in VTA dopamine (*Slc6a3)* and GABA (*Gad1)* neurons. **A-B** Representative 10x (**A**) and 63x (**B**) images of the VTA and substantia nigra labelled with mRNAscope probes for *Hcn1, Slc6a3, and Gad1. Hcn1* co-expression was observed in *Gad1*$^{+}$ neurons but not in *Slc6a3*$^{+}$ neurons. n = 4 rats.

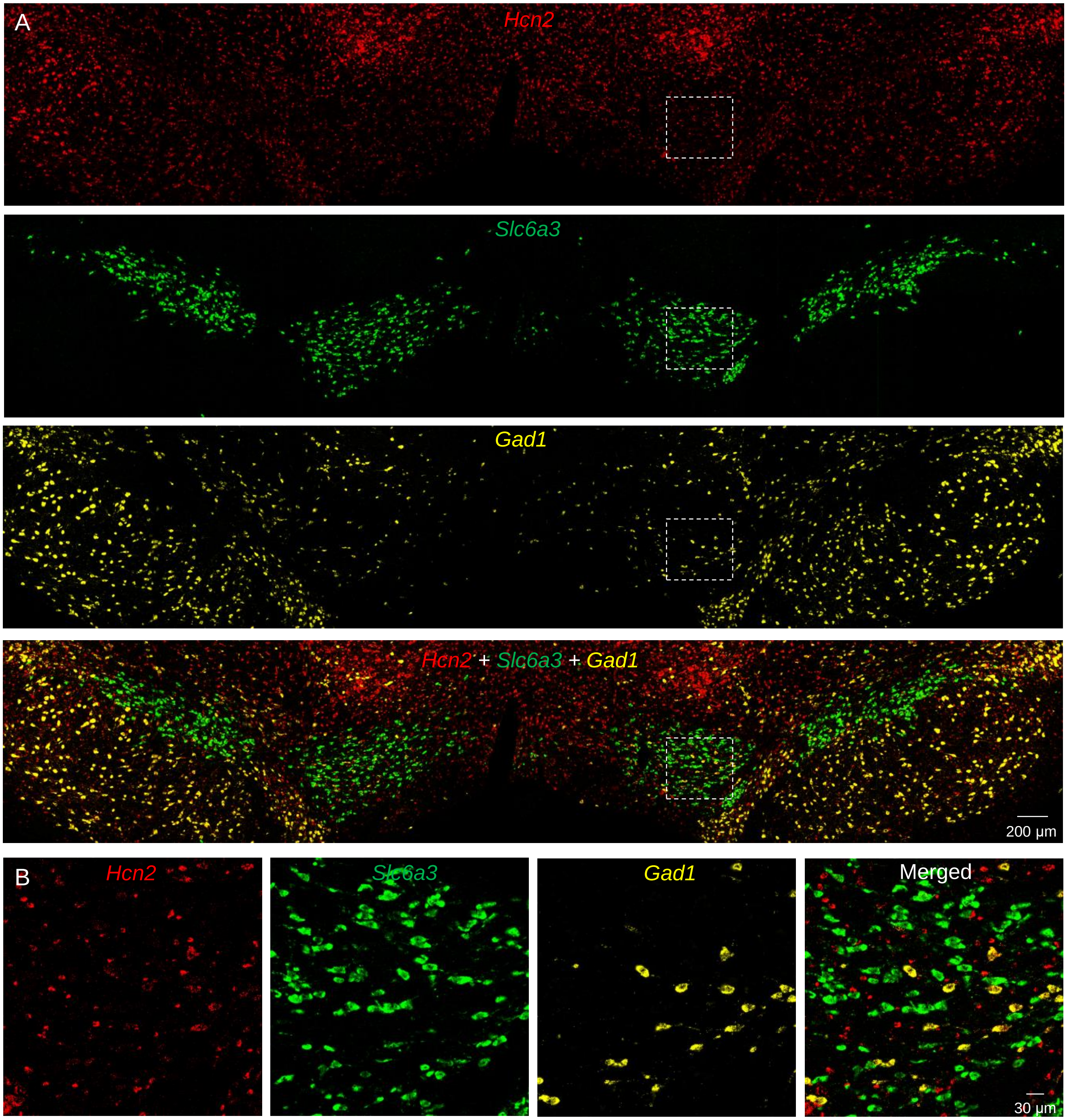

Figure S2. Expression of *Hcn2* mRNA in VTA dopamine (*Slc6a3)* and GABA (*Gad1)* neurons. **A-B** Representative 10x (**A**) and 63x (**B**) images of *Hcn1* mRNA in the VTA and substantia nigra. *Hcn2* co-expression was observed in *Gad1*$^{+}$ neurons but not in *Slc6a3*$^{+}$ neurons. n = 4 rats.

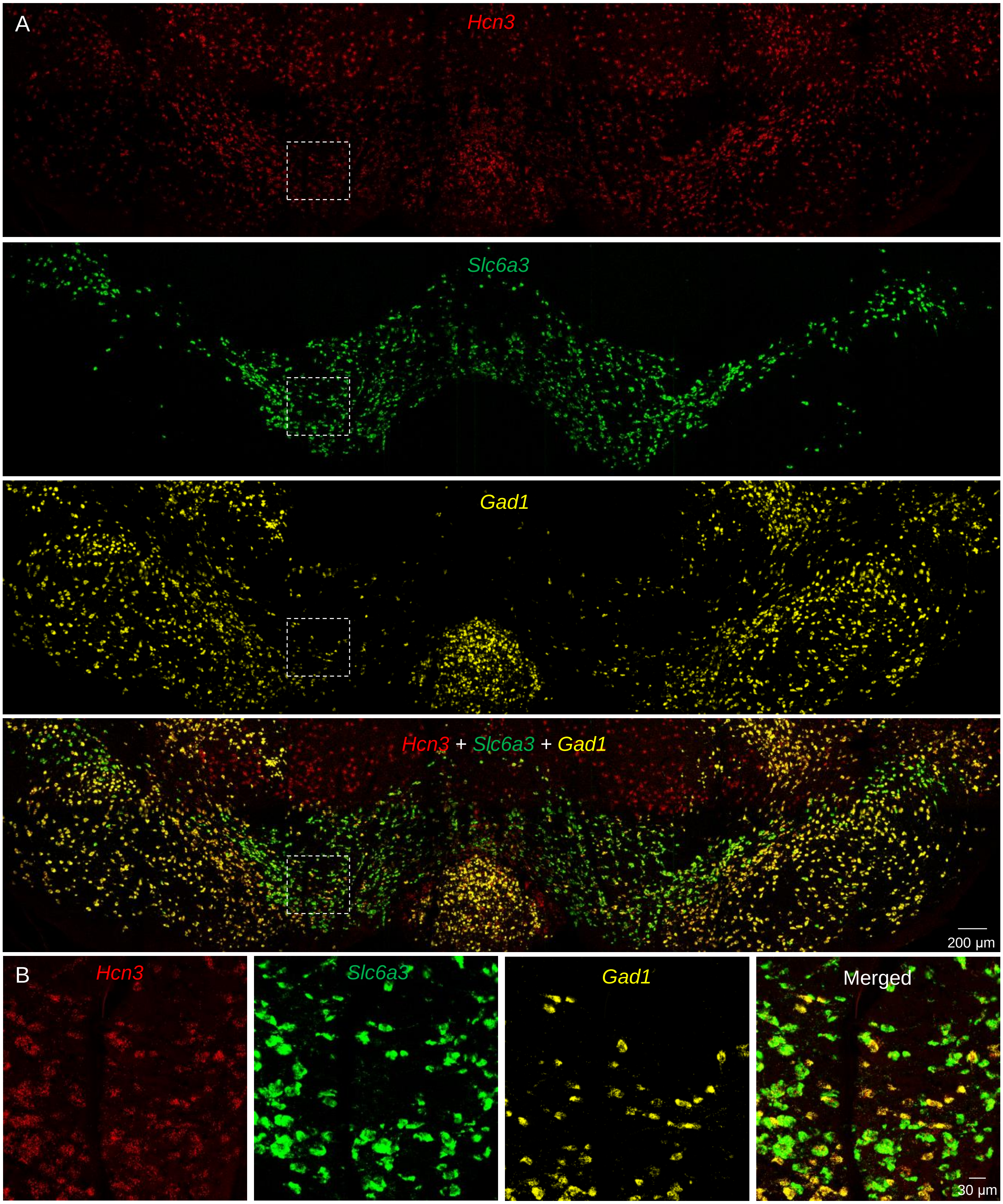

Figure S3. Expression of *Hcn3* mRNA in VTA dopamine (*Slc6a3)* and GABA (*Gad1)* neurons. **A-B** Representative 10x (**A**) and 63x (**B**) images of *Hcn3* mRNA is expressed in the VTA and substantia nigra. *Hcn3* is co-expressed in both *Gad1*$^{+}$ neurons and *Slc6a3*$^{+}$ neurons. n = 4 rats.

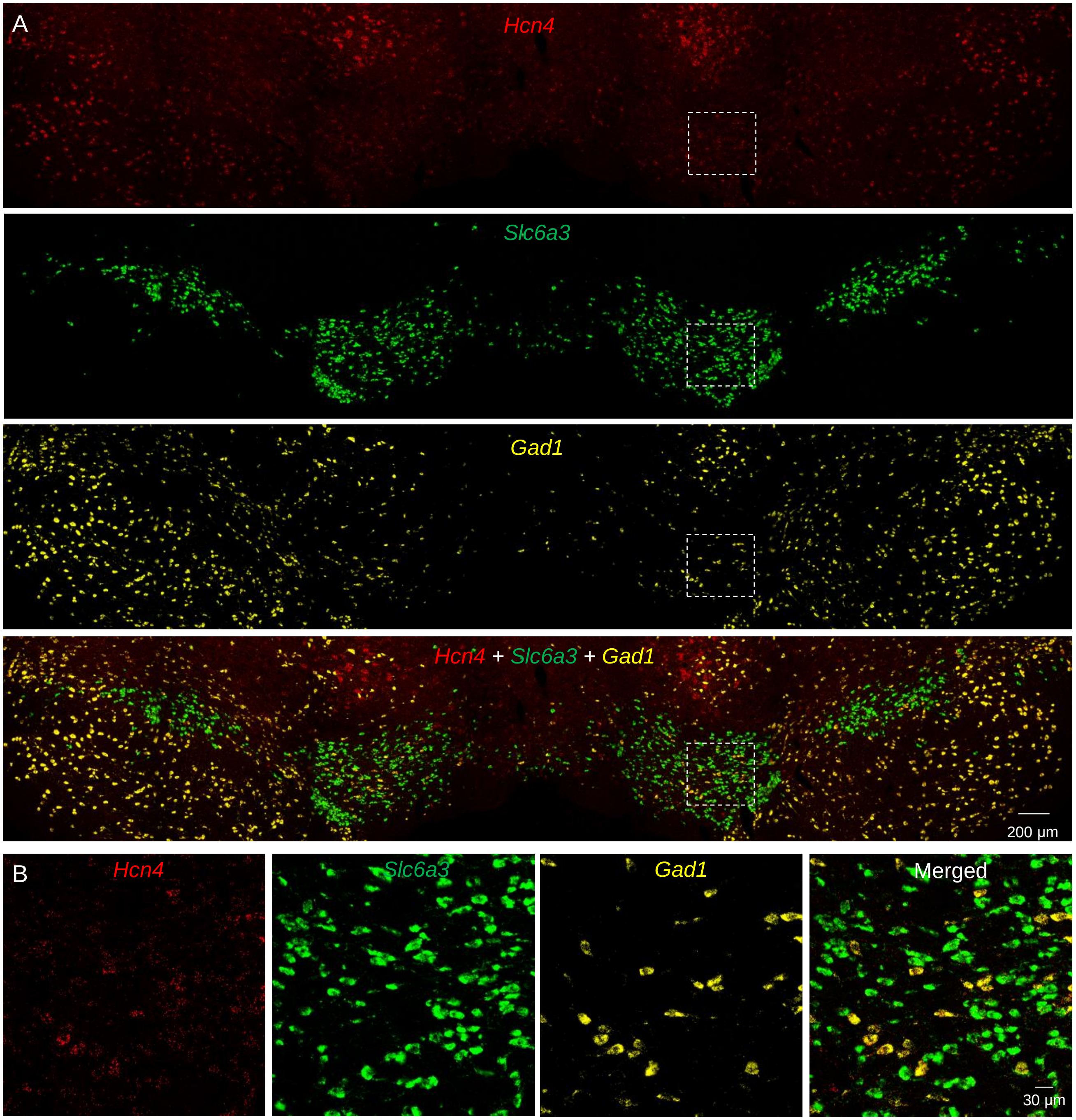

Figure S4. Expression of *Hcn4* mRNA in VTA dopamine (*Slc6a3)* and GABA (*Gad1)* neurons. **A-B** Representative 10x (**A**) and 63x (**B**) images of *Hcn4* mRNA is expressed in the VTA and substantia nigra. *Hcn4* is co-expressed in both *Gad1*$^{+}$ neurons and *Slc6a3*$^{+}$ neurons. n = 4 rats.

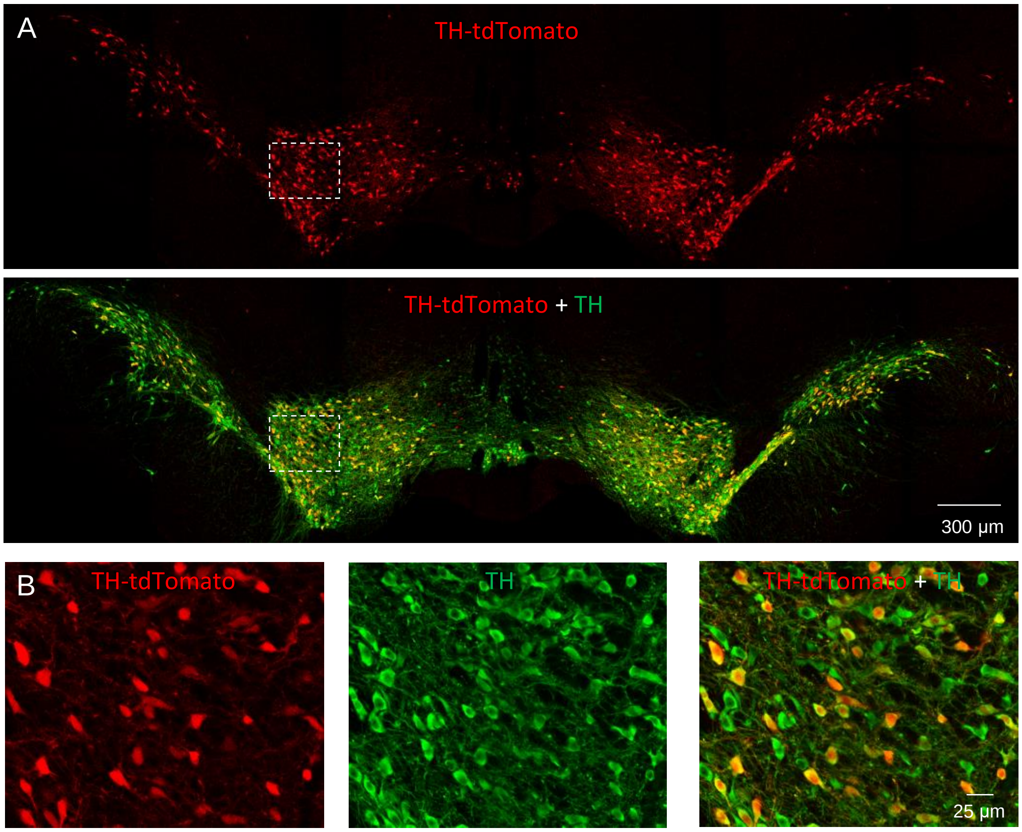

Figure S5. Immunohistochemistry showing expression of tdTomato on dopamine (TH+) neurons of TH-tdTomato reporter rats. **A-B** Representative 10x (**A**) and 25x (**B**) magnification images showed that tdTomato was expressed on dopamine neurons in VTA and substantia nigra pars compacta (SNc). tdTomato was co-localized with 64.7 ± 1.8% of TH^{+} neurons in both the VTA and SNc. n = 6 rats.

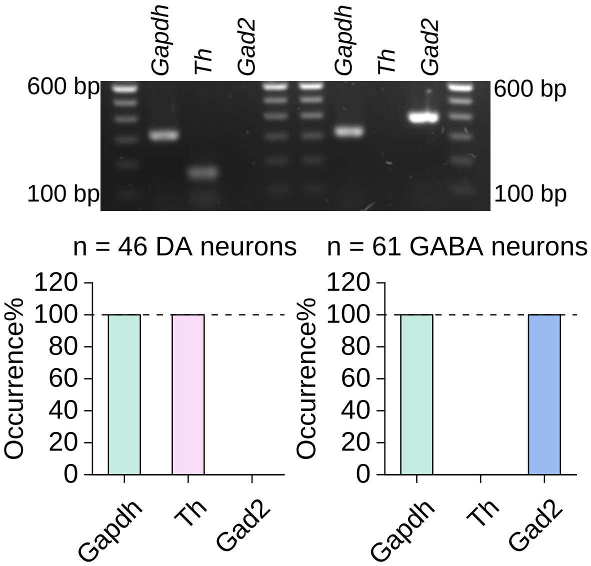

Figure S6. Single-cell RT PCR verification of VTA neuronal cell types after electrophysiological recordings. In all the TH-tdTomato negative neurons recorded, 46 were *Th* expression dopamine (DA) neurons, 61 were *Gad2* expression GABA neurons.

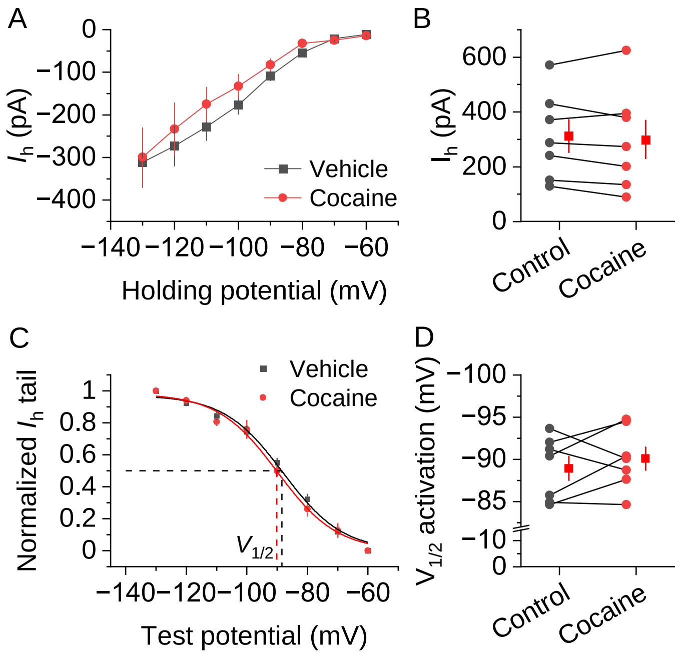

Figure S7. A-D Bath application of cocaine (10 μM) did not significantly change I_h amplitude (**A, B**; *t*-test, t_{12} = 0.1, p = 0.896) and $V_{1/2}$ (**C,D**; *t*-test, t_{12} = 0.6, p = 0.563) in VTA GABA neurons.

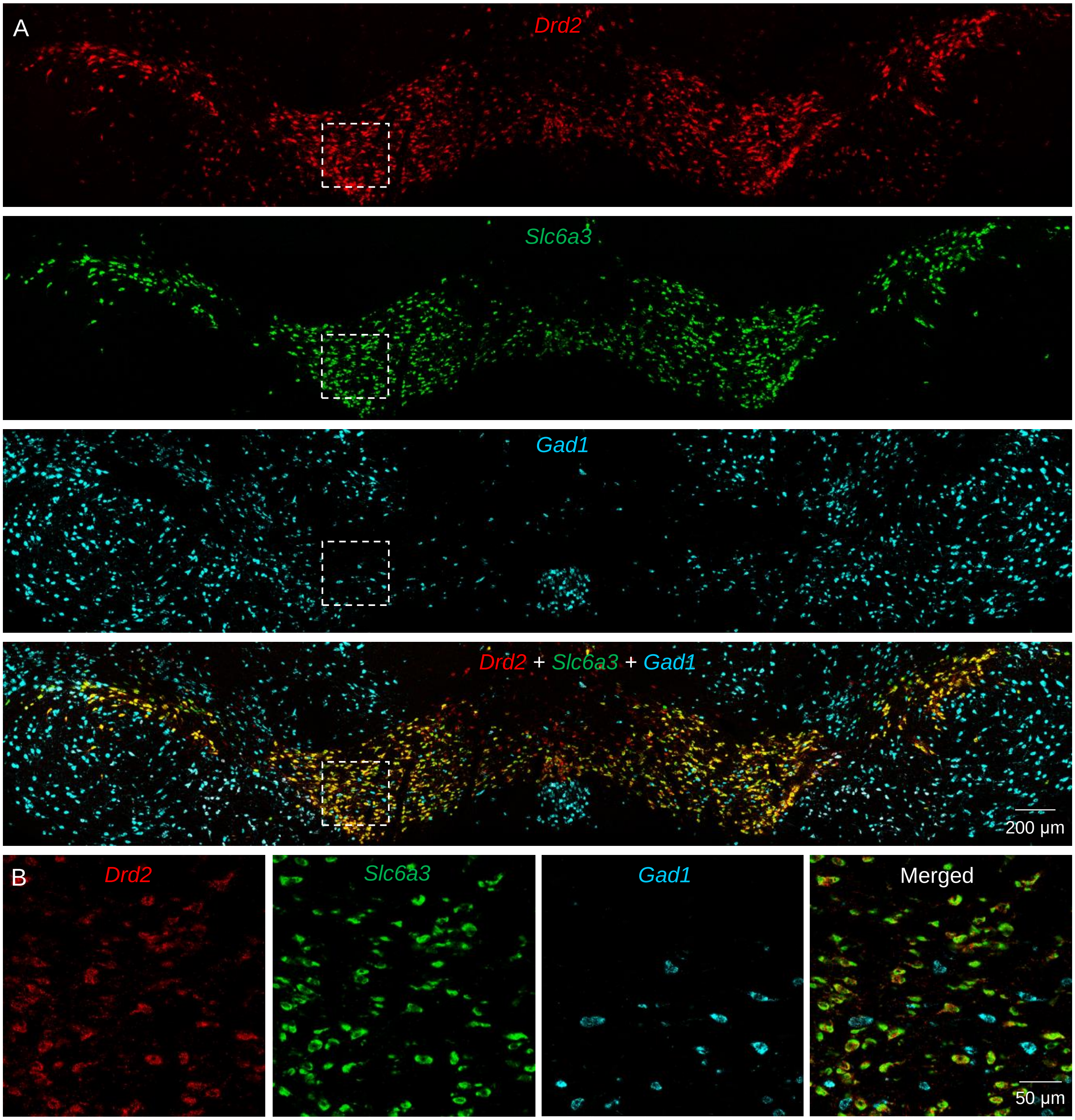

Figure S8. *Drd2* mRNA was expressed in all dopamine neurons (*Slc6a3)* but was only ~11% of GABA neurons (*Gad1)* in the VTA. **A-B** Representative 10x (**A**) and 63x (**B**) images of *Drd2* mRNA was expressed in the VTA and substantia nigra. n = 2 rats.

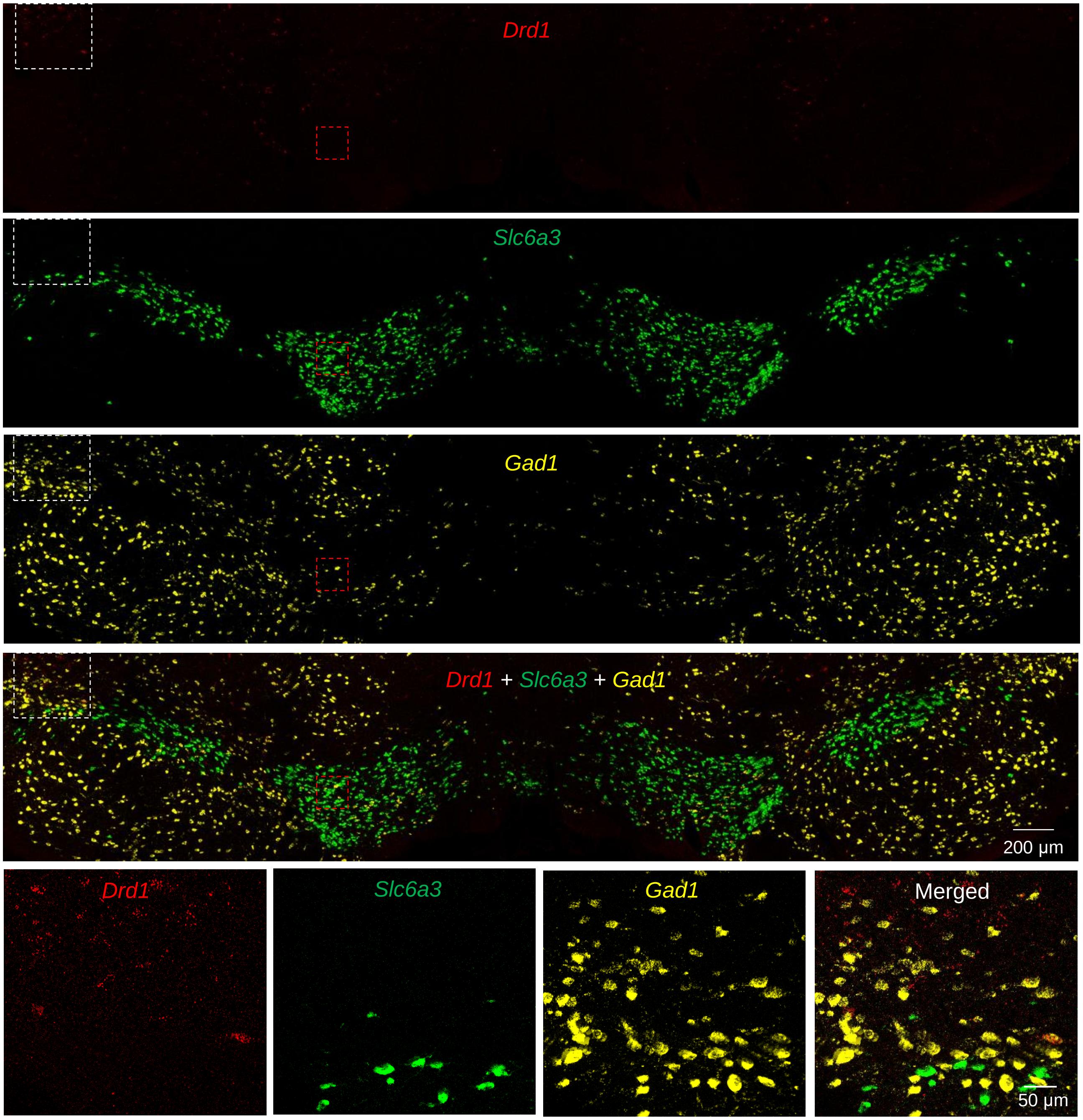

Figure S9. The expression of *Drd1* mRNA in the midbrain. *Drd1* mRNA was barely detected in in the VTA but were detected in regions neighboring to the VTA (white dash boxes). Red dash boxes denotes the expression of *Drd1* mRNA in VTA in Fig. 3G. n = 2 rats.

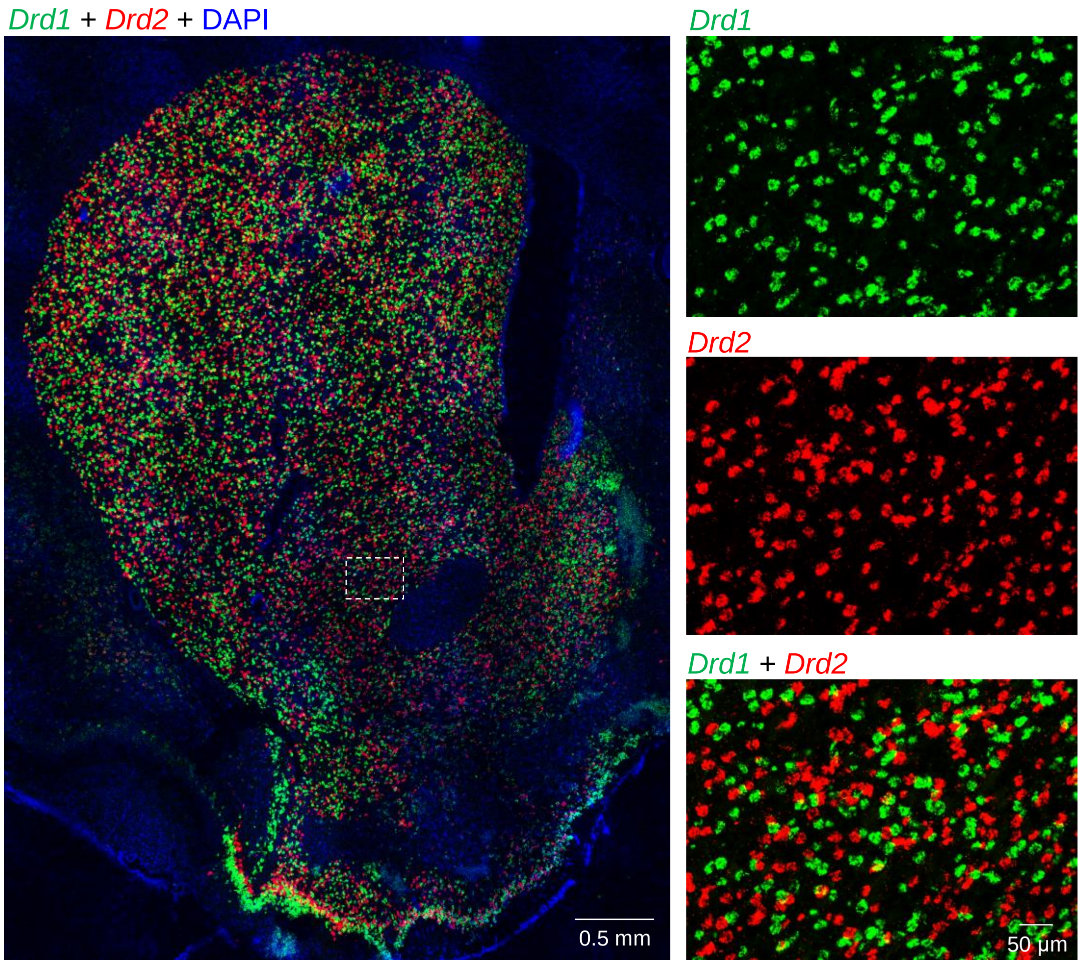

Figure S10. Representative 10x (left) and 63x (right) images of *Drd1 and Drd2* mRNA expression in the striatum. n = 2 rats.

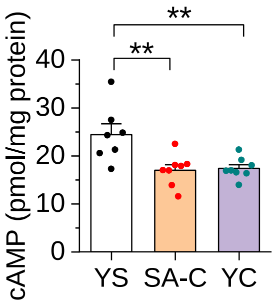

Figure S11. ELISA shows that cAMP levels were decreased in the VTA in rats following cocaine self-administration (SA-C) and yoked cocaine administration (YC) compared with yoked saline (YS) administration (one-way ANOVA, $F_{2,20} = 8.2$, $p = 0.003$). n = 7-8 rats for each group, $^{**}p < 0.01$ for Tukey's post hoc tests.

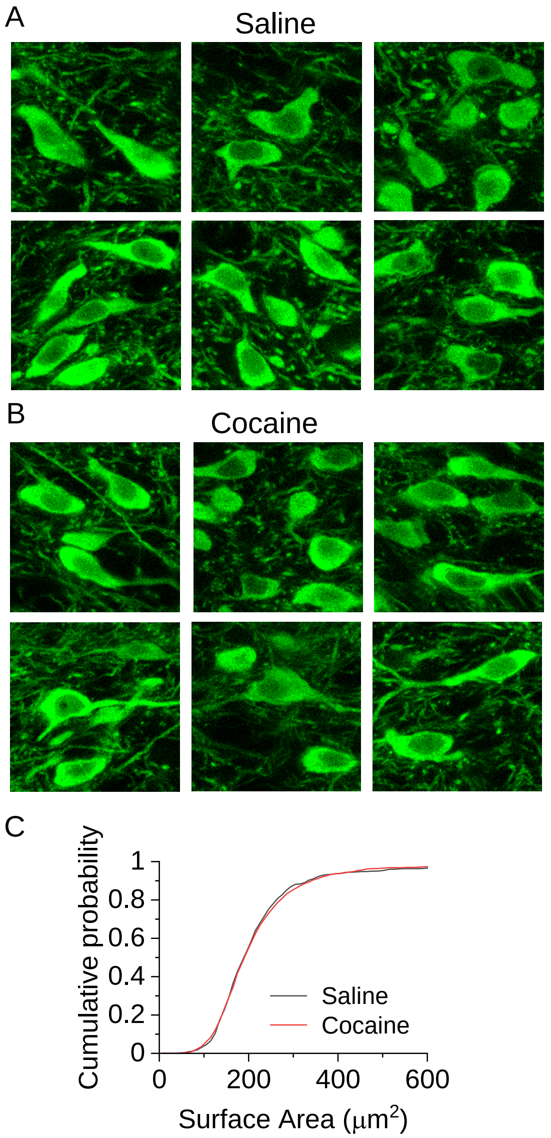

Figure S12. Cocaine self-administration did not significantly change the size of dopamine neurons in VTA. **A** Representative fixed midbrain sections with TH staining from rats received 10 days of yoked-saline infusions. **B** Representative midbrain sections from rats received 10 days of cocaine self-administration labeled with TH staining. **C** Cumulative distributions of VTA dopamine neuron soma surface area. Saline: n = 915 neurons from three rats; Cocaine: n = 1296 neurons from three rats; $p > 0.05$, Kolmogorov–Smirnov tests.

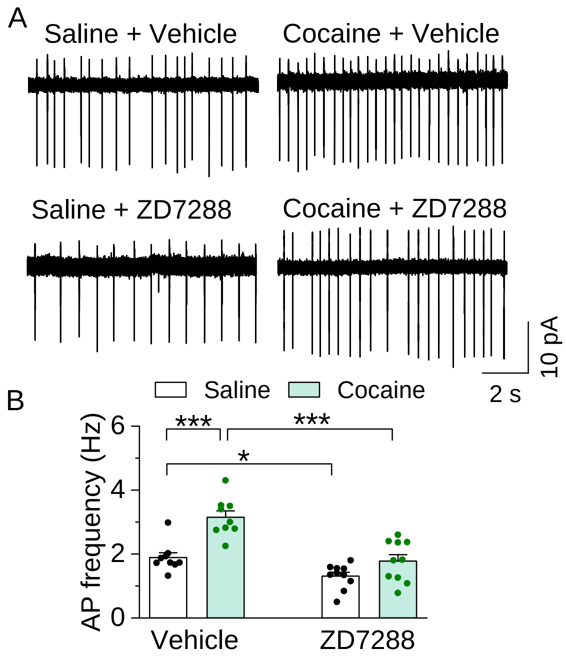

Figure S13. Cocaine self-administration-induced I_h upregulation may contribute to the increase in the frequency AP firing of VTA dopamine neurons. **A** Representative AP firing recorded in a cell-attached mode in TH-tdTomato rats that received cocaine self-administration or yoked saline administration. ZD7288 (20 µM) or vehicle was perfused into the ACSF. **B** Cocaine self-administration and ZD7288 had significant main effects on the frequency of AP firing (two-way ANOVA, cocaine, $F_{1,34} = 25.1$, $p = 0.001$; ZD7288, $F_{1,34} = 31.9$, $p < 0.001$; cocaine x ZD7288 interaction, $F_{1,34} = 5.2$, $p = 0.028$). Tukey's *post hoc* analysis revealed that cocaine self-administration significantly increased the frequency of AP firing (*$p = 0.023$), while ZD7288 significantly reduced the frequency of AP firing in both cocaine and saline groups (***$p < 0.001$). n = 9-10 neurons from 3-4 rats in each group).

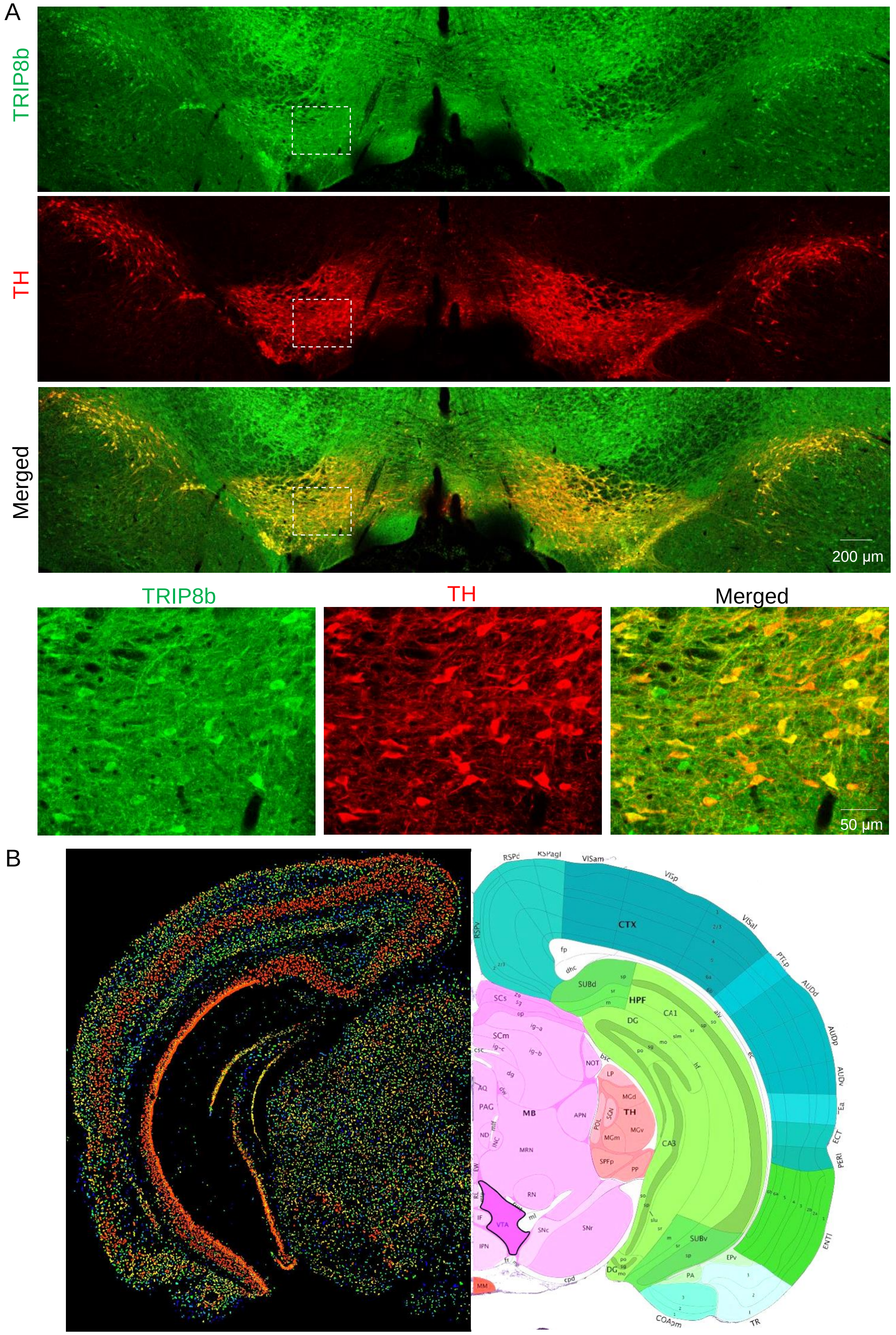

Figure S14. Expression of TRIP8b in the midbrain including VTA dopamine neurons. **A** Immunohistochemistry shows that TRIP8b is widely expressed in the midbrain and is co-localized with TH^+ in the VTA (n = 3 rats). **B** mRNA expression data of TRIP8b (*Pex5i*) from Allen Brain Atlas (mouse.brain-map.org). The intensity is color-coded to range from blue (low expression intensity), through green (medium intensity) to red (high intensity). TRIP8b mRNA is enriched in the VTA and is expressed in medium to high intensity.

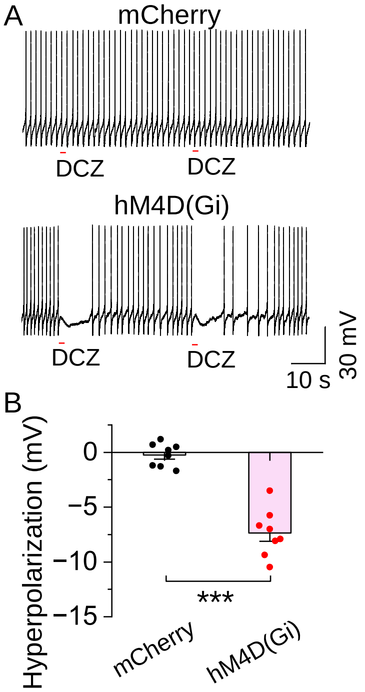

Figure S15. A,B Pressure ejection of DCZ (1 µM) resulted in hyperpolarization and a pause of AP firing in hM4D(Gi)-expressing VTA dopamine neurons but had no impact on AP firing in mCherry-expressing neurons (*t*-test, $t_{14} = 8.4$, $p < 0.001$).

A

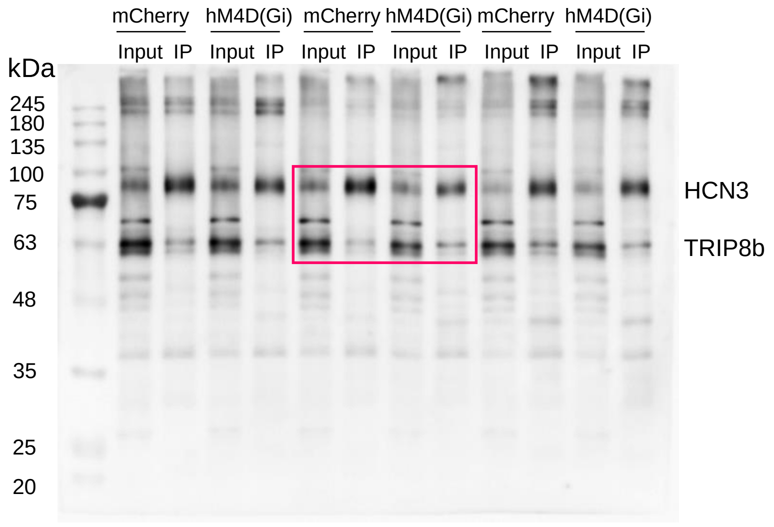

IP: anti-HCN3
IB: anti-TRIP8b, anti-HCN3

B

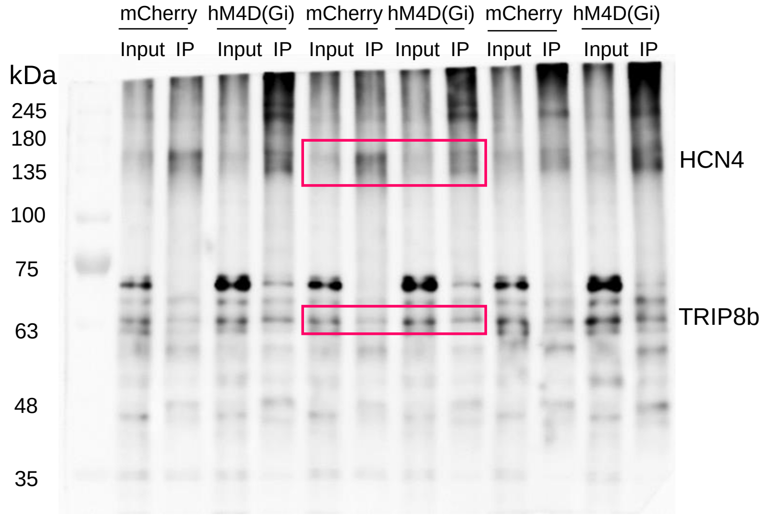

IP: anti-HCN4
IB: anti-TRIP8b, anti-HCN4

Figure S16. Uncropped blot corresponding to Fig. 6J,K. **A** Co-immunoprecipitation was carried out using anti-HCN3 antibodies and VTA lysate from rats that expressed hM4D(Gi) compared with rats that expressed the control vector mCherry following chronic yoked DCZ administration. **B** Same as described in A except HCN4 antibodies were used. ‘Input’ refers to the original VTA lysate and ‘IP’ refers to the protein eluted from beads. Red boxes denote the cropped image presented in Fig. 6J,K.

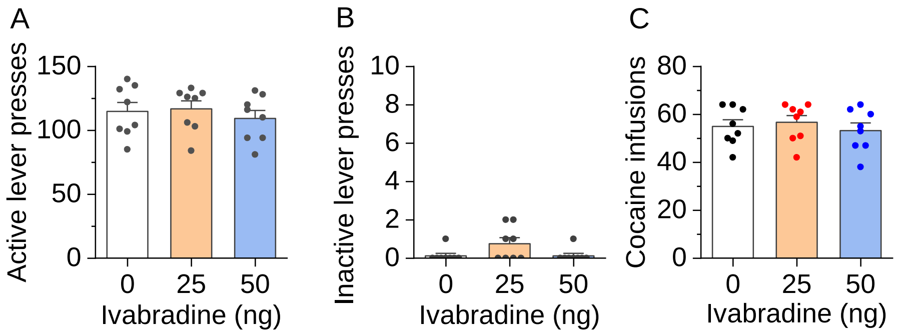

Figure S17. A-C Intra-VTA ivabradine microinjection did not alter the active lever presses (**A**; one-way ANOVA, $F_{2,21} = 0.4$, $p = 0.700$) and inactive lever presses (**B**; one-way ANOVA, $F_{2,21} = 3.0$, $p = 0.071$) of rats under FR2 reinforcement. **C** Ivabradine pretreatments did not significantly alter cocaine infusions attained under FR2 reinforcement (One-way ANOVA, $F_{2,21} = 0.3$, $p = 0.723$, n = 8 rats in each group).

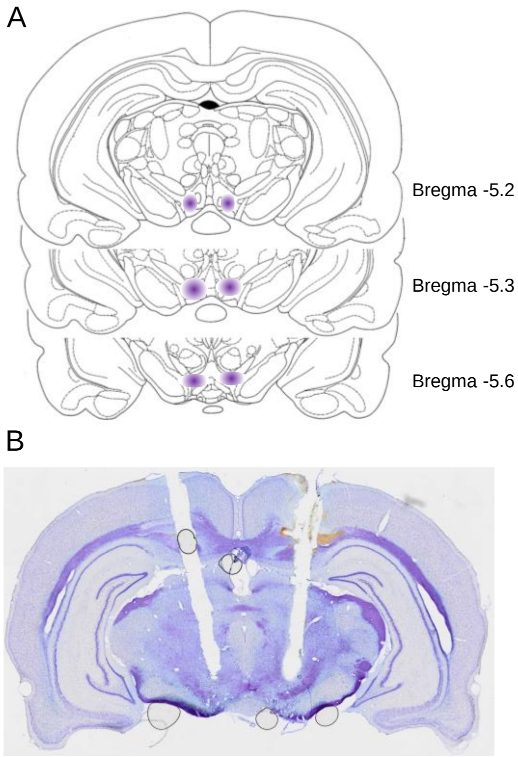

Figure S18. Verification of intra-VTA cannula placements after self-administration. **A** Representative sites of cannula tips in the VTA of rats. **B** Cresyl Violet-stained coronal section of typical intra-VTA cannula placement.

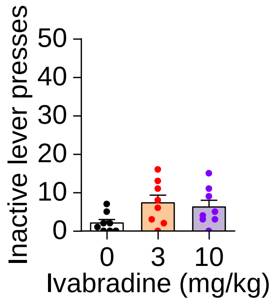

Figure S19. Systemic ivabradine administration (0, 3, 10 mg/kg, i.p.) did not alter the inactive lever presses (one-way ANOVA: $F_{2,23}$ = 3.1, p = 0.067) under FR2 reinforcement schedule (n = 8 rats in each group).

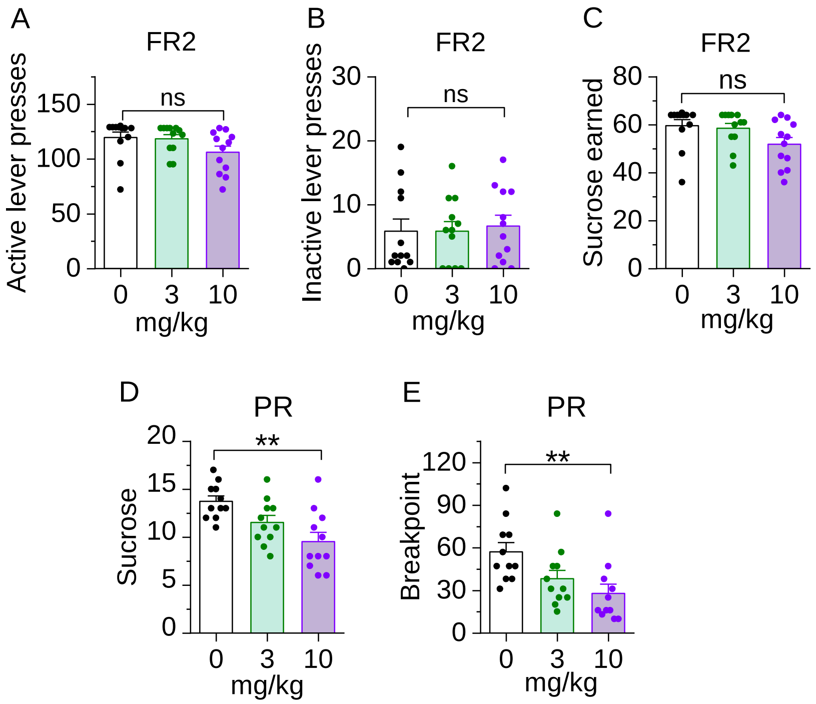

Figure S20. Systemic ivabradine administration reduced oral sucrose self-administration under a PR schedule but did not affect sucrose intake under a FR2 schedule. **A,B,C** Ivabradine (3, 10 mg/kg, i.p.) did not affect the number of active lever presses (one-way ANOVA: $F_{2,33}$ = 2.3, p = 0.113) and inactive lever presses ($F_{2,33}$ = 0.1, p = 0.923) and sucrose pellets earned ($F_{2,33}$ = 2.8, p = 0.073; n = 12 rats). ns, not significant. **D,E** Ivabradine produced a dose-dependent reduction of sucrose pellets earned (one-way ANOVA: $F_{2,30}$ = 7.6, p = 0.002) and breakpoint (Kruskal-Wallis one-way ANOVA on ranks: H_2 = 10.4, p = 0.005) under a PR schedule under FR2 reinforcement schedule (n = 11 rats).
